# GLIS3: A novel transcriptional regulator of mitochondrial functions and metabolic reprogramming in postnatal kidney and polycystic kidney disease

**DOI:** 10.1016/j.molmet.2024.102052

**Published:** 2024-11-05

**Authors:** Justin B. Collier, Hong Soon Kang, Yun-Gil Roh, Chitrangda Srivastava, Sara A. Grimm, Alan K. Jarmusch, Anton M. Jetten

**Affiliations:** 1 Cell Biology Group, Immunity, Inflammation and Disease Laboratory, National Institute of Environmental Health Sciences, National Institutes of Health, Research Triangle Park, NC, 27709, USA; 2 Integrative Bioinformatics, National Institute of Environmental Health Sciences, National Institutes of Health, Research Triangle Park, NC, 27709, USA; 3 Metabolomics Core Facility, Immunity, Inflammation and Disease Laboratory, National Institute of Environmental Health Sciences, National Institutes of Health, Research Triangle Park, NC, 27709, USA

**Keywords:** GLIS3, Metabolic reprogramming, Transcription, Oxidative phosphorylation, Polycystic kidney disease, Aerobic glycolysis

## Abstract

****Objectives**:**

Deficiency in the transcription factor (TF) GLI-Similar 3 (GLIS3) in humans and mice leads to the development of polycystic kidney disease (PKD). In this study, we investigate the role of GLIS3 in the regulation of energy metabolism and mitochondrial functions in relation to its role in normal kidney and metabolic reprogramming in PKD pathogenesis.

**Methods:**

Transcriptomics, cistromics, and metabolomics were used to obtain insights into the role of GLIS3 in the regulation of energy homeostasis and mitochondrial metabolism in normal kidney and PKD pathogenesis using GLIS3-deficient mice.

**Results:**

Transcriptome analysis showed that many genes critical for mitochondrial biogenesis, oxidative phosphorylation (OXPHOS), fatty acid oxidation (FAO), and the tricarboxylic acid (TCA) cycle, including *Tfam*, *Tfb1m*, *Tfb2m*, *Ppargc1a*, *Ppargc1b, Atp5j2*, *Hadha*, and *Sdha,* are significantly suppressed in kidneys from both ubiquitous and tissue-specific *Glis3-*deficient mice. ChIP-Seq analysis demonstrated that GLIS3 is associated with the regulatory region of many of these genes, indicating that their transcription is directly regulated by GLIS3. Cistrome analyses revealed that GLIS3 binding loci frequently located near those of hepatocyte nuclear factor 1-Beta (HNF1B) and nuclear respiratory factor 1 (NRF1) suggesting GLIS3 regulates transcription of many metabolic and mitochondrial function-related genes in coordination with these TFs. Seahorse analysis and untargeted metabolomics corroborated that mitochondrial OXPHOS utilization is suppressed in GLIS3-deficient kidneys and showed that key metabolites in glycolysis, TCA cycle, and glutamine pathways were altered indicating increased reliance on aerobic glycolysis and glutamine anaplerosis. These features of metabolic reprogramming may contribute to a bioenergetic environment that supports renal cyst formation and progression in *Glis3*-deficient mice kidneys.

**Conclusions:**

We identify GLIS3 as a novel positive regulator of the transition from aerobic glycolysis to OXPHOS in normal early postnatal kidney development by directly regulating the transcription of mitochondrial metabolic genes. Loss of GLIS3 induces several features of renal cell metabolic reprogramming. Our study identifies GLIS3 as a new participant in an interconnected transcription regulatory network, that includes HNF1B and NRF1, critical in the regulation of mitochondrial-related gene expression and energy metabolism in normal postnatal kidneys and PKD pathogenesis in *Glis3*-deficient mice.

## Highlights

• GLIS3 is identified as a new regulator of the bioenergetic changes during early postnatal kidney development.
• Loss of GLIS3 function suppresses metabolic and mitochondrial genes and promotes features of metabolic reprogramming.
• Mitochondrial OXPHOS metabolism is decreased and aerobic glycolysis increased in *Glis3*-deficient kidneys.
• GLIS3 regulates metabolic and mitochondrial gene transcription likely in coordination with HNF1B and NRF1.

## 1 Introduction

In addition to the pancreas, thyroid gland, and testis, the Krüppel-like zinc finger transcription factor (TF) GLI-Similar 3 (GLIS3) plays a critical role in the regulation of gene transcription and biological functions in the kidney [[1], [2], [3]]. Loss of function mutations in human *GLIS3* are causally linked to a syndrome characterized by neonatal diabetes and congenital hypothyroidism (NDH) that is accompanied by the development of PKD [2,4]. GLIS3-deficient mice (*Glis3*-KO2) mimic the pathology observed in humans, including the development of PKD [2,3,5,6].

During mouse embryonic kidney development Glis3 mRNA expression is first observed in comma and S-shaped bodies; however, GLIS3 does not appear to have a major role in regulating ureteric bud branching and early nephrogenesis [1,5]. At later stages, GLIS3 is expressed in parietal cells, and epithelial in the proximal and distal tubules, loop of Henle, and collecting ducts. The development of PKD in GLIS3-deficient mice, as well as in kidney-specific knockout mice, is characterized by the formation of glomerular cysts, dilation of proximal tubules, and cyst formation in distal tubules and collecting ducts [5,7]. Postnatally, renal cysts steadily increase in size and number leading to a gradual loss of normal kidney function, including a decrease in estimated glomerular filtration rate [5,7]. These studies highlight the critical role for GLIS3 in maintaining normal kidney functions. However, our understanding of the renal processes and functions regulated by GLIS3 and its role in PKD pathogenesis is still very limited.

Development and progression of PKD are associated with changes in several metabolic processes involving various interconnected signaling and transcription regulatory pathways [[8], [9], [10], [11], [12], [13], [14], [15], [16]]. A metabolic shift from aerobic glycolysis to oxidative phosphorylation is an integral part of normal early postnatal kidney development, while dysregulation of these energy metabolic pathways in renal cells, referred to as metabolic reprogramming, is a well-recognized feature of acute and chronic kidney diseases, including PKD [13,[17], [18], [19], [20]]. However, relatively little is known about the transcriptional control of these changes in energy homeostasis.

We hypothesized that GLIS3 might play a role in the regulation of the metabolic shift during early postnatal kidney development and that this may involve transcriptional regulation of mitochondria-related and metabolic genes by GLIS3. To investigate this, we analyzed how lack of GLIS3 function affected the expression of metabolic and mitochondria-related genes and assessed how these transcriptional changes relate to alterations in energy homeostasis and mitochondrial functions in ubiquitous and kidney-specific *Glis3-*deficient mice. Transcriptome analysis of kidneys from wild type (WT) and *Glis3-*deficient mice revealed that lack of GLIS3 represses the upregulation of many mitochondria-related genes, including OXPHOS- and TCA cycle-related genes, normally observed during early postnatal kidney development. GLIS3 cistrome analysis demonstrated that GLIS3 directly regulates the transcription of several of these genes by binding to GLIS binding sites (GLISBS) in their promoter and/or enhancer regions. We further provide evidence indicating that GLIS3 regulates the transcription of many of these genes in coordination with hepatocyte nuclear factor 1 beta (HNF1B) and nuclear respiratory factor 1 (NRF1). Seahorse and untargeted metabolomic analyses demonstrated that the observed alterations in gene expression in *Glis3*-deficient kidneys resulted in changes in cellular metabolism, including suppressed utilization of mitochondrial OXPHOS, and increased aerobic glycolysis and altered glutamine anaplerosis.

In this study, we provide new insights into the molecular mechanism that controls the shift from aerobic glycolysis to OXPHOS during the first month of postnatal kidney development using *Glis3*-deficient mice. We identify GLIS3, together with HNF1B and NRF1, as components of an interconnected transcriptional regulatory network that plays a critical role in the control of mitochondrial-related gene regulation and energy metabolism in normal kidneys and PKD pathogenesis. Kidneys of *Glis3*-deficient mice exhibit several features of metabolic reprogramming, a bioenergetic environment that contributes to abnormal renal cell proliferation and progression of renal cyst formation [19,21]. Our study suggests that GLIS3 has a protective role against cyst formation and that increased expression or activation of GLIS3 may have potential for future therapeutic strategies.

## 2 Material and methods

Other details for materials and methods are presented in the Supplementary Materials.

### 2.1 Mice

Ubiquitous Glis3-deficient *Glis3*-KO2 mice (C57BL/6-Glis3<tm3(mCherry)Amj>), *Glis3*-EGFP mice (C57BL/6-Glis3<tm3(Glis3-EGFP)Amj>) expressing a GLIS3-EGFP fusion protein were described previously [3]. Tissue-selective *Glis3*-Pax8Cre mice (B6; 129-Glis3<tm2Amj> Pax8<tm1.1(cre)Mbu>) were generated by crossing Glis3^fl/fl^ mice with Pax8Cre mice (B6.129P2(Cg)-Pax8^tm1.1(cre)Mbu^/J; Jackson Laboratory # 028196). Mice were backcrossed onto C57BL/6 for at least seven generations. Mice were routinely fed an NIH-31 diet (ND; Harlan) and euthanized using CO_2_. For *Glis3*-KO2 and *Glis3*-Pax8Cre mice experiments, WT littermates/age-matched mice were used as controls. All animal studies followed guidelines outlined by the NIH Guide for the Care and Use of Laboratory Animals and protocols were approved by the Institutional Animal Care and Use Committee at the NIEHS.

### 2.2 RNA-seq and ChIP-Seq data analysis

RNA-Seq was performed as described previously [2,3]. RNA-seq data from kidneys of PND7, PND14, and PND28 *Glis3*-KO2 and *Glis3*-Pax8Cre mice and littermate controls for each strain were used for the mitochondrial gene expression analysis. To analyze GLIS3 and HNF1B binding to mitochondrial-related genes, we performed ChIP-Seq analysis as described previously [3]. Briefly, kidney cells were dissociated whole kidneys from *Glis3*-EGFP mice for GLIS3 and from WT mice for HNF1B at PND7 mice then were cross-linked in 1% formaldehyde for 10 min and the reaction subsequently quenched by the addition of 125 mM glycine for 10 min. The cross-linked cells were washed two times with PBS, resuspended in lysis buffer A for 10 min, pelleted, and resuspended in lysis buffer B for 10 min. Samples were subsequently sheared in lysis buffer C for 40 min using an S220 focused-ultrasonicator (Covaris, Woburn, MA). After centrifugation, the cleared chromatin supernatant was incubated with an anti-GFP (ab290; Abcam) or anti-HNF1B (#720259; ThermoFisher) antibody for ChIP. After subsequent washes, ChIPed-DNA was eluted and amplified. Libraries were synthesized using a NEXTflex Rapid DNA-Seq kit (PerkinElmer, Austin, TX). Sequencing was performed with NextSeq 500 or MiSeq (Illumina, San Diego, CA). RNA-Seq and ChIP-Seq analysis data for the study were deposited under accession numbers GSE240532, GSE240074, and GSE240072. For the comparison of GLIS3 and HNF1B binding with that of NRF1, NRF1 ChIP-Seq data from GSE136532 (ENCODE Consortium (https://doi.org/10.1038/nature11247) was used.

### 2.3 Primary renal epithelial cell (REC) isolation

Two-week-old WT and *Glis3*-KO2 mice were euthanized, and kidneys immediately removed and placed in ice-cold PBS. Kidneys were thoroughly minced using an X-ACTO knife and placed into conical tubes of ice-cold digestion buffer consisting of 0.5 mg/mL type 1 collagenase and 0.25 mg/mL soybean trypsin inhibitor (ThermoFisher) dissolved in Hank’s Balanced Salt Solution. The tubes were placed in a 37 °C water bath and shaken for approximately 15 min. Digestion was followed by 2 min gravity sedimentation, the top layer was subsequently transferred to a clean tube and digestion quenched by addition of FBS and centrifuged at 200 g for approximately 5 min. This process was repeated two times with the final resuspension using growth culture media consisting of advanced DMEM/F-12 Flex Media (ThermoFisher) supplemented with 1x MEM amino acids, 1x ITS-X, 0.5 mM sodium pyruvate, 2 mM glutamax, 10 mM glucose, 2% FBS, and 1% penn-strep (ThermoFisher) and plated onto 6- or 12-well plates. RECs isolated from 1 mouse is *n* = 1. Primary RECs were grown to 80–90% confluency and directly used or after one passage.

### 2.4 Statistical analysis and other software

All data are shown as mean ± S.E.M. When comparing two experimental groups, an unpaired, two-tailed t-test was used to determine statistical differences. A two-way analysis of variance (ANOVA) followed by Tukey’s post hoc test was performed for comparisons of multiple groups. *p* < 0.05 was considered statistically significant unless otherwise specified. All statistical tests were performed using GraphPad Prism software (GraphPad Software, San Diego, CA). BioRender.com online application was used for creating illustrations.

## 3 Results

### 3.1 Mitochondrial OXPHOS-related genes are repressed in Glis3-KO2 kidneys

**Figure 1:**
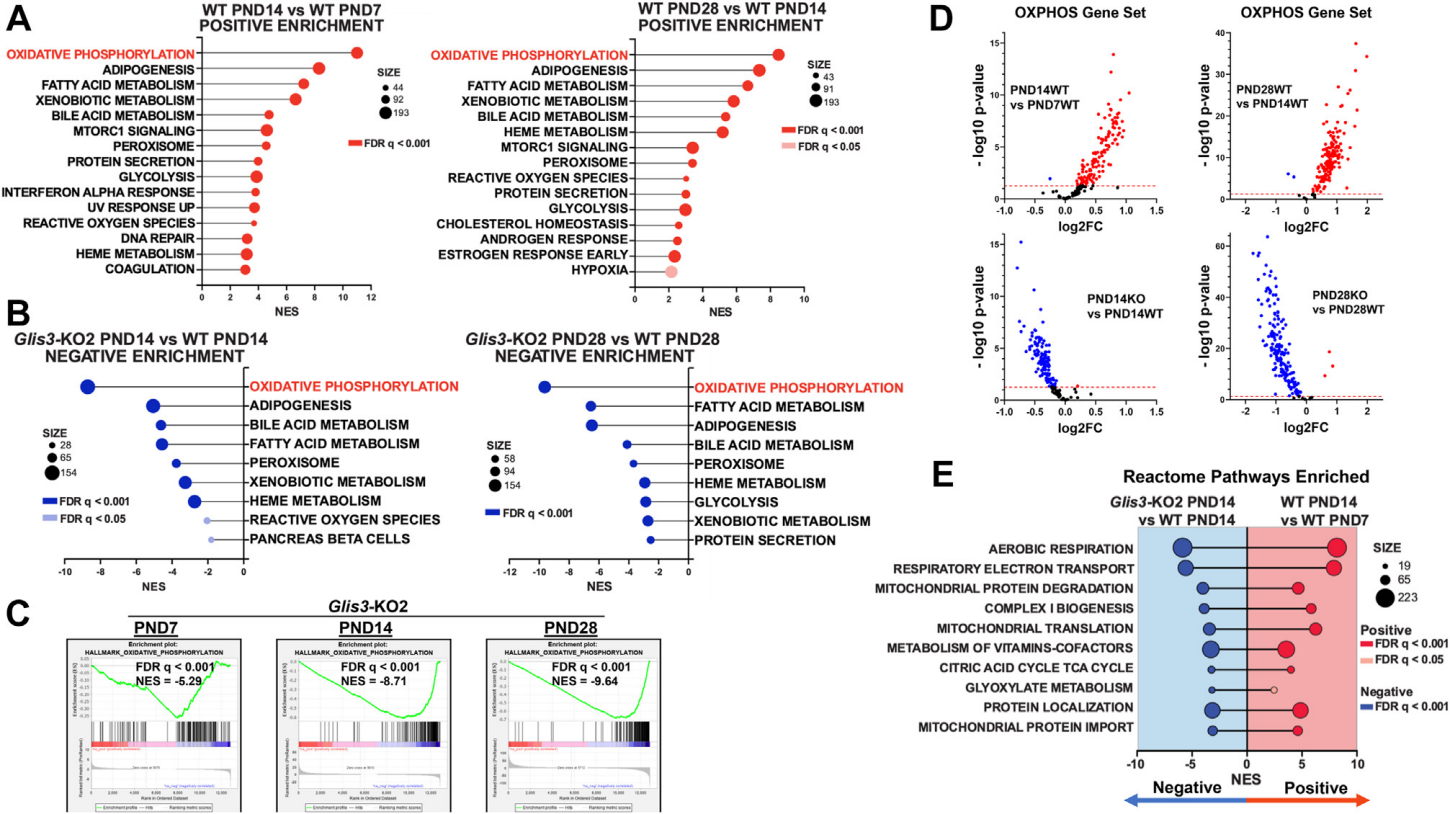
**Mitochondrial-related pathways are suppressed in *Glis3-*KO2 kidneys.** (A) GSEA Hallmark pathway showing the top positively enriched age-associated pathways for WT kidneys ranked by the normalized enrichment score (NES) and showing FDR and gene set size. The topmost enriched pathways are in red. (B) GSEA Hallmark pathway showing the top negatively enriched age-associated pathways for *Glis3*-KO2 kidneys ranked by NES and showing FDR and gene set size. The topmost enriched pathways are in blue. (C) GSEA OXPHOS enrichment plots showing negative enrichment in the *Glis3-*KO2 kidneys at PND7, 14, and 28. (D) Volcano plots of all Hallmark OXPHOS genes showing upregulation with age (red) in the WT PND14 vs WT PND7 and WT PND28 vs WT PND14 and downregulation of the OXPHOS gene set (blue) in *Glis3-*KO2 PND14 vs WT PND14 and *Glis3*-KO2 vs WT PND28 kidneys. (E) GSEA pathway analysis depicting the top enriched Reactome pathways in the WT PND14 vs WT PND7 compared to the Glis3-KO2 PND14 vs WT PND14 showing opposite enrichment. For WT the pathways show positive enrichment, and for the Glis3-KO2 showing negative enrichment. Reactome pathways ranked by NES in the Glis3-KO2 PND14, and showing FDR and gene set size. (For interpretation of the references to color in this figure legend, the reader is referred to the Web version of this article.)

Changes in mitochondrial functions and energy metabolism are well-known aspects of normal postnatal kidney maturation, while mitochondrial dysfunction is a key element of many renal pathologies [13,18,19]. To investigate whether loss of GLIS3 function affects these processes during postnatal kidney development and whether GLIS3 plays a role in the transcriptional regulation of genes associated with mitochondrial functions, we analyzed the gene expression profiles of kidneys from WT and *Glis3-*KO2 mice at PND7, 14, and 28 by RNA-Seq analysis. Gene set enrichment analysis (GSEA) of WT kidney RNA-seq data identified oxidative phosphorylation (OXPHOS) as the most positively enriched Hallmark pathway at PND14 compared to PND7, and similarly at PND28 compared to PND14 (Figure 1A). This is consistent with previous reports showing a transition towards mitochondrial metabolism during early postnatal kidney development [13,18,19]. In contrast, OXPHOS was the most negatively enriched GSEA Hallmark pathway in *Glis3-*KO2 kidneys compared to WT (Figure 1B) at all three time points with the differences increasing with age (Figure 1C). Volcano plots of the Hallmark OXPHOS gene set, the top enriched pathway, shows that most (97%) of the OXPHOS genes up-regulated at PND14 and PND28 in WT kidneys were suppressed in PND14 and PND28 *Glis3-*KO2 kidneys (Figure 1D). These data indicate that loss of GLIS3 function suppresses the increase in OXPHOS gene expression normally observed during postnatal kidney development. This was supported by GSEA Reactome pathway analysis demonstrating that as WT kidneys mature from PND7 to PND14, key mitochondria-related processes, including aerobic respiration, electron transport, complex I biogenesis, mitochondrial translation, and the TCA cycle were positively enriched, while these same pathways in the *Glis3-*KO2 kidneys were significantly negatively enriched (Figure 1E). This was consistent with GSEA Gene Ontology component analysis showing that genes related to mitochondrial components were negatively enriched in *Glis3-*KO2 kidneys (Supplementary Fig. S1). Together, these analyses indicated that loss of GLIS3 function suppressed the increase in mitochondrial metabolism normally observed during the first few weeks of postnatal kidney development.

**Figure 2:**
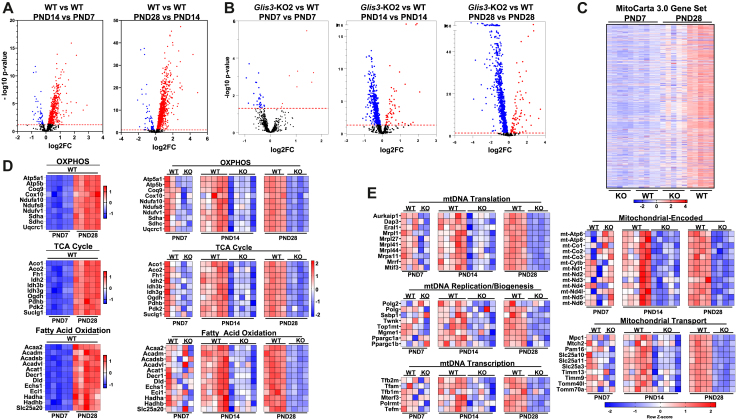
**The expression of mitochondria-related genes is decreased in *Glis3-*KO2 kidneys.** (A) Visualization of differential gene expression in WT kidneys. Volcano plots of DEGS of WT PND14 vs WT PND7 and WT PND28 vs WT PND14 kidney RNA-seq data against the MitoCarta 3.0 gene dataset. (B) Visualization of differential gene expression in *Glis3*-KO2 kidneys. Volcano plots of DEGS of PND7, 14, and 28 *Glis3-*KO2 and WT kidney RNA-seq data against the MitoCarta 3.0 gene dataset. (C) Heatmap of the MitoCarta 3.0 gene set in alphabetical order. Gene expression is compared between PND7 and PND28 WT and *Glis3-*KO2. Upregulated genes are represented in red and downregulated genes in blue. Expression values are shown as z-scores of the rlog-transformed values for each gene. (D, E) Heatmaps of DEGs in *Glis3-*KO2 versus WT kidneys at PND7, 14, and 28 labeled with mitochondria-associated pathways. For OXPHOS, TCA Cycle, and FAO a heat map of WT PND28 vs WT PND7 is included showing the increase in expression with age. Upregulated genes are represented in red and downregulated genes in blue. Expression values are shown as z-scores of the rlog-transformed values for each gene. (For interpretation of the references to color in this figure legend, the reader is referred to the Web version of this article.)

Differentially expressed genes (DEGs) in *Glis3-*KO2 kidneys (compared to WT kidneys) were subsequently analyzed using the MitoCarta 3.0 database representing 1140 mouse mitochondria-related genes [22]. Volcano plots of the MitoCarta gene set show that expression of these genes in WT kidneys progressively increased between PND7-14 and from PND14-28 (Figure 2A). In contrast, the number of downregulated mitochondria-related genes, as well as the fold-difference in their expression, increased progressively in *Glis3*-KO2 kidneys during PND7–28 (Figure 2B). This analysis further showed that most genes up-regulated in WT kidneys were suppressed in *Glis3*-KO2 kidneys consistent with the GSEA OXPHOS analysis shown in Figure 1D. This was supported by heatmap analysis of the MitoCarta gene set (Figure 2C), which demonstrated that while the loss of GLIS3 function greatly suppressed the induction of most genes, it did not completely block the normal upregulation of MitoCarta genes during PND7-28 (Figure 2C). The extent of these changes in mitochondrial metabolic gene expression during PND7–28 paralleled the progression of cystogenesis observed in H&E-stained sections of *Glis3*-KO2 kidneys (Supplementary Fig. S2). Lists of mitochondria- and metabolism-related genes differentially expressed in *Glis3-*KO2 kidneys, including OXPHOS, TCA cycle, fatty acid oxidation (FAO), and mitochondrial biogenesis associated genes, are presented in Supplementary Tables S1–4. The heatmaps in Figure 2D display the temporal expression pattern of individual genes related to mitochondrial metabolic pathways during the first month of postnatal kidney development, including genes associated with OXPHOS (e.g., *Atp5a*, *Ndufs8*), the TCA cycle (e.g., *Idh2*, *Pkd2*), and FAO (e.g., *Acadm*, *Hadha*). It shows the upregulation of these genes in WT kidneys between PND7-PND28 and their suppression in *Glis3*-KO2 kidneys. The temporal and differential expression of mitochondrial-encoded genes (e.g., *mt-Atp6*, *mt–Co1*), mitochondrial transport (e.g., *Mtch2*, *Tomm70a*), replication (e.g., *Polg*, *Twnk*), transcription (e.g., *Tfam*, *Tfb1/2m, Polrmt*), and translation (e.g., *Mrpl1*, *Mrrf*), are shown in Figure 2E. The heatmaps are consistent with the conclusion that during PND7-28 differences in mitochondrial metabolic gene expression between WT and *Glis3*-KO2 kidneys (compare differences in red vs blue intensities at each age) became increasingly larger. Transcriptome analysis further demonstrated that loss of GLIS3 function repressed both nuclear- and mitochondrial-encoded genes. The downregulation of the expression of several nuclear-encoded genes, including *Tfam*, *Tfb2m*, *Tfb1m, Ppargc1a*, and *Polrmt,* with known critical roles in the transcriptional regulation of mitochondrial biogenesis [[23], [24], [25]], suggested that GLIS3 may act upstream of these TFs and regulate the expression of certain mitochondria-related genes indirectly. This is consistent with previous reports showing that GLIS3 is not expressed in mitochondria.

**Figure 3:**
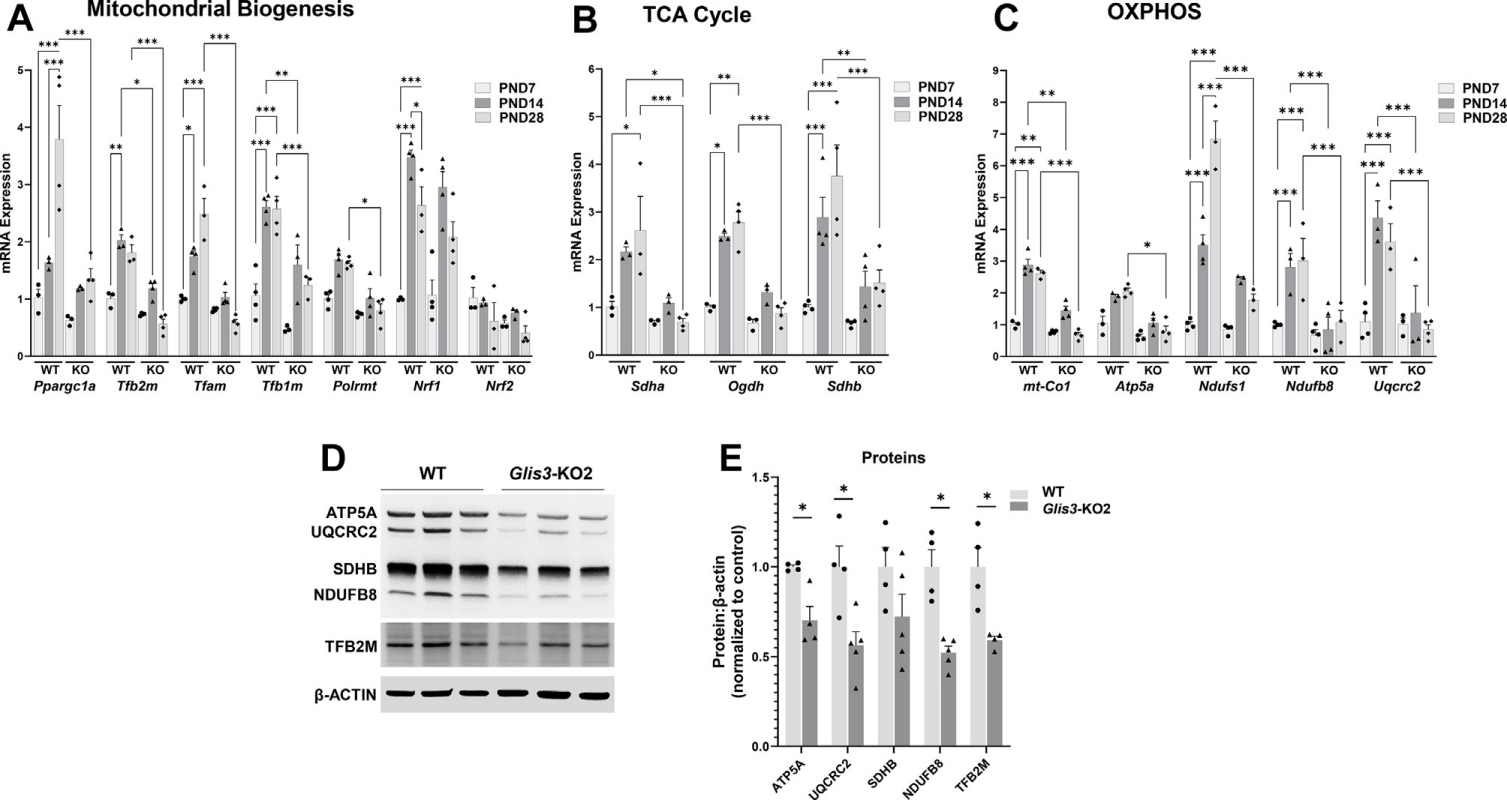
**GLIS3 is required for the upregulation of mitochondria-related gene expression during early postnatal kidney development.** (A–C) RT-qPCR analysis of the expression of several genes related to mitochondrial biogenesis, TCA cycle, and OXPHOS in PND7, 14, and 28 WT and *Glis3*-KO2 kidneys. Data are represented as mean ± SEM, *n* ≥ 3. ∗∗∗ represents *p* < 0.001; ∗∗ represents *p* < 0.01; ∗ represents *p* < 0.05. (D–E) Representative immunoblot of ATP5A, UQCRC2, SDHB, NDUFB8, and TFB2M protein expression in WT and *Glis3-*KO2 kidneys. Protein expression was quantified by densitometric analysis. Data are represented as mean ± SEM, *n* ≥ 4. ∗ Represents *p* < 0.05.

The increase in mitochondrial-related gene expression during early postnatal kidney development in WT mice and their suppression in *Glis3-*KO2 kidneys was supported by RT-qPCR analysis. The expression of the mitochondrial biogenesis regulatory genes, *Ppargc1a*, *Tfam*, *Tfb2m*, and *Tfb1m* (Figure 3A), several OXPHOS and TCA cycle-related genes, including *Sdha/b*, *Ogdh*, *Ndufs1, Ndufb8, and Uqcrc2*, and the mitochondria encoded genes, *mt–Co1* and *Atp5a1*, increased in WT kidneys from PND7 to 14 or PND7 to 28, while these increases were suppressed in *Glis3-*KO2 kidneys (Figure 3B,C). At PND28, the level of expression of many of these genes was 65–75% lower in *Glis3-*KO2 kidneys compared to WT kidneys. The reduced expression of *Tfb2m*, *Atp5a1*, *Ndufb8*, and *Uqcrc2* RNA in *Glis3-*KO2 kidney was associated with decreased levels of the corresponding protein (Figure 3D,E). The suppression of *Tfb2m,* as well as that of *Tfam* in *Glis3-*KO2 mice, was observed only in kidneys and not in other tissues tested, such as the heart, liver, pancreas, and islets, suggesting that this phenotype is kidney-specific (Supplementary Fig. S3).

**Figure 4:**
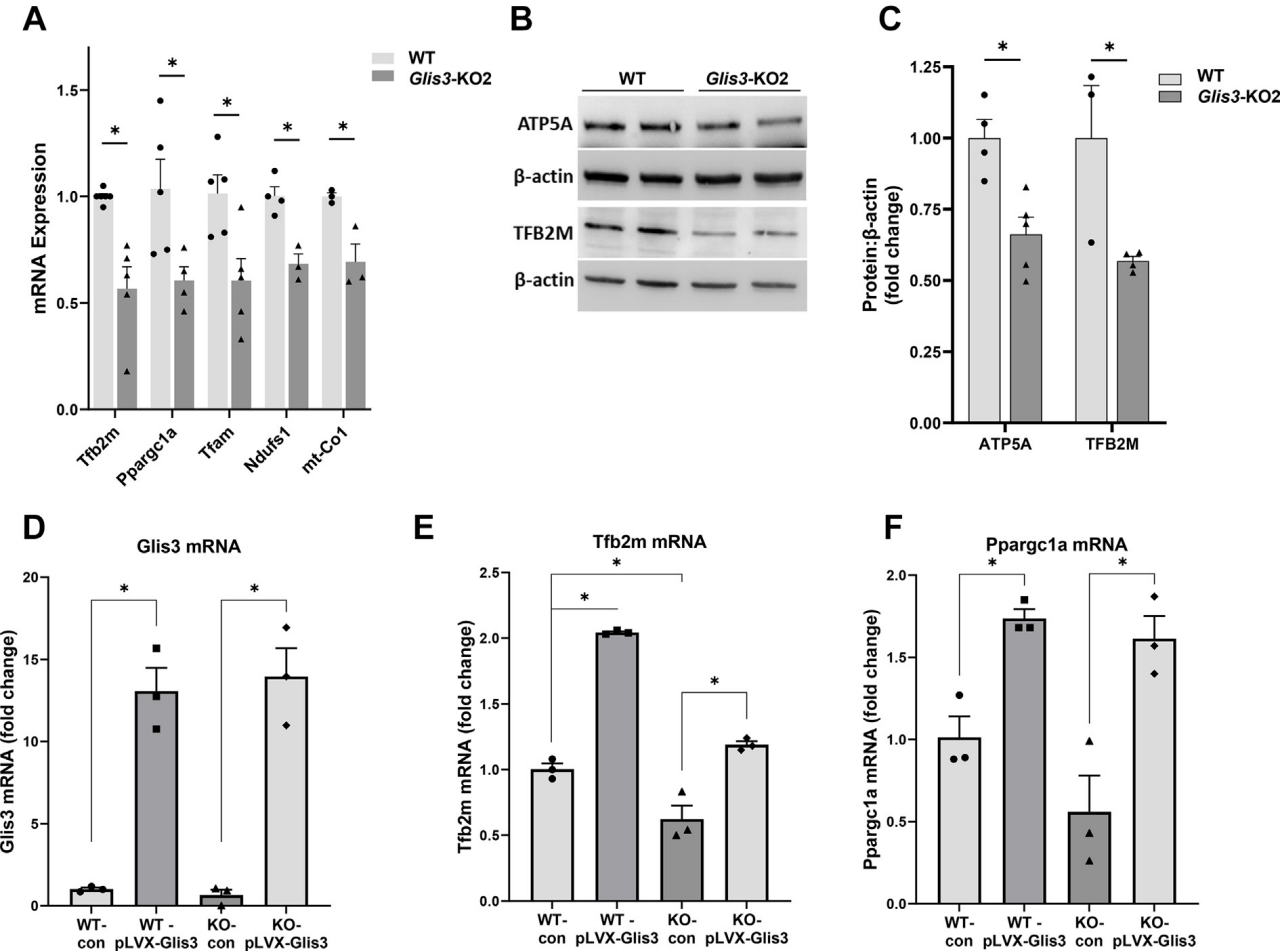
**Suppression of mitochondria-related gene expression in primary *Glis3-*KO2 RECs and increased expression by exogenous GLIS3.** (A) RT-qPCR analysis of the expression of mitochondria-related genes in WT and *Glis3-*KO2 RECs. Data are represented as mean ± SEM, *n* ≥ 3. (B) Immunoblot analysis of ATP5A and TFB2M expression in WT and KO primary RECs. (C) Densitometric analysis of ATP5A and TFB2M proteins in WT and KO primary RECs. Data are represented as mean ± SEM, *n* ≥ 4. (D) Overexpression of GLIS3 in WT and *Glis3-*KO2 RECs increased *Tfb2m* and *Ppargc1a* mRNA expression. RECs were infected with Glis3 lentivirus for 36 h and gene expression analyzed by RT-qPCR. Data are represented as mean ± SEM, *n* = 3. ∗ Represents *p* < 0.05.

Analysis of gene expression in primary cultures of renal epithelial cells isolated from WT and *Glis3-*KO2 mouse kidneys supported the decreased expression of mitochondria-related genes, including *Tfb2m*, *Ppargc1a*, *Tfam, Ndufs1,* and *mt–Co1* in *Glis3-*KO2 cells (Figure 4A). The decreased *Tfb2m* and *Atp5a* mRNA expression in cultured *Glis3*-KO2 renal epithelial cells was associated with a reduction in corresponding protein levels (Figure 4B,C). The regulation of mitochondrial metabolic genes by GLIS3 was further supported by data showing that expression of exogenous GLIS3 (Figure 4D) caused an increase in *Tfb2m* and *Ppargc1a* mRNA levels in both WT and *Glis3*-KO2 primary renal epithelial cells (Figure 4E,F).

### 3.2 Transcriptional regulation of mitochondria-related genes by GLIS3

**Figure 5:**
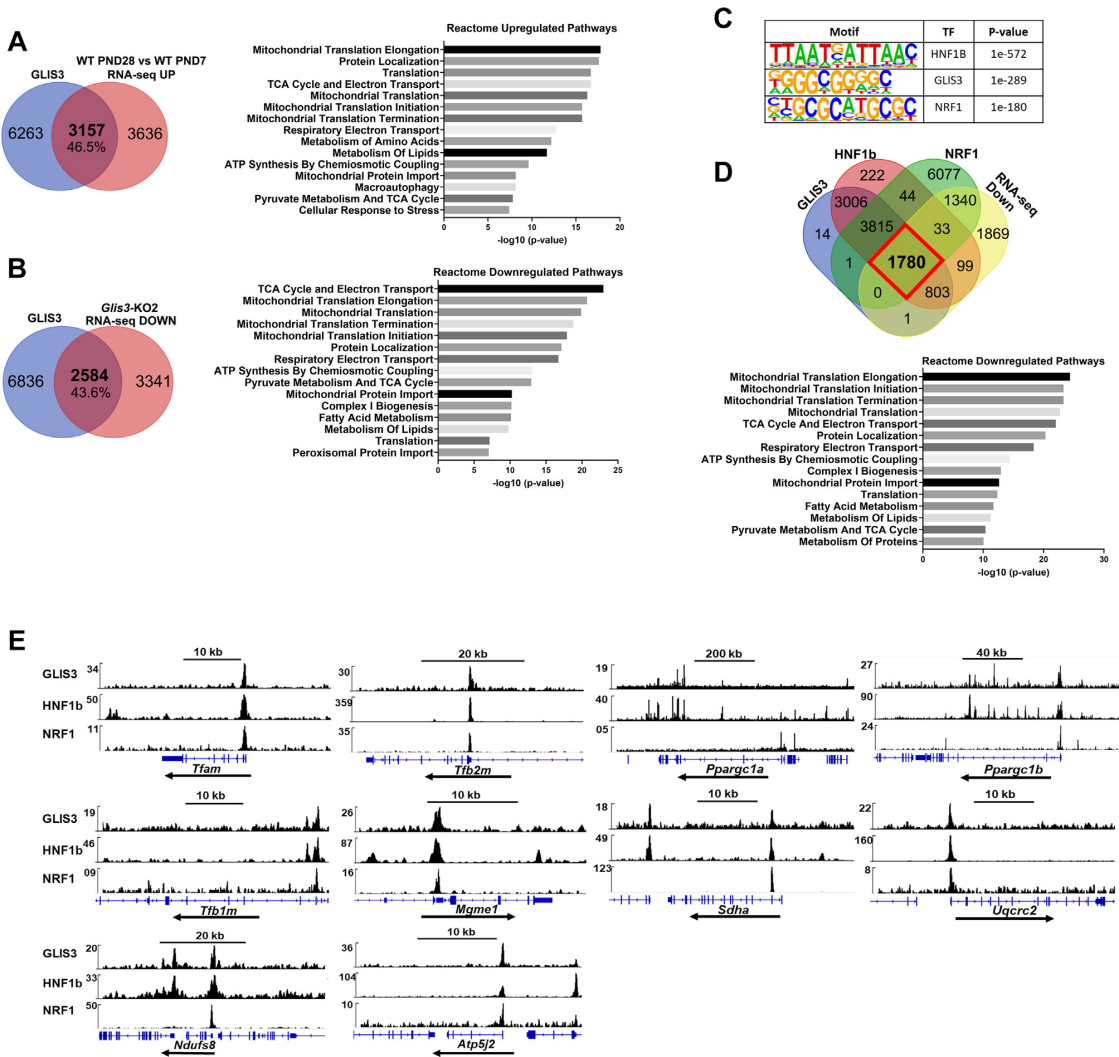
**GLIS3 regulates several metabolic and mitochondria-related genes in mouse kidneys in coordination with HNF1B and NRF1.** (A) Venn diagram showing that GLIS3 was bound to 46.5% of the genes upregulated between PND7-28 in WT kidneys. Reactome pathway analysis of this gene set identified mitochondrial/biogenesis-related genes as the topmost significantly up-regulated pathways. Venn diagram was generated with PND7 GLIS3 ChIP-seq and WT PND28 vs WT PND7 RNA-seq datasets. GLIS3 binding peaks within 5 kb from TSS. (B) Venn diagram showing that GLIS3 was bound to 43.6% of the genes suppressed in PND28 *Glis3-*KO2 kidneys compared to WT kidneys. Reactome pathway analysis of this gene set identified mitochondrial/biogenesis-related genes as the topmost significantly down-regulated pathways. Venn diagram was generated with GLIS3 ChIP-seq and PND28 *Glis3-*KO2 vs WT RNA-seq datasets. (C) HOMER *de novo* motif analysis of GLIS3 ChIP-Seq data identified the GLIS3 binding site (GLISBS) as a top binding site among consensus binding sequences. HNF1B and NRF1 binding sequences are frequently localized near GLISBS. (D) Venn diagram showing overlaps between genes downregulated in *Glis3-*KO2 kidneys and genes with GLIS3, HNF1B, and NRF1 peaks (at 5 kb from TSS) (outlined in red in diagram). Reactome pathway analysis of the 1780 overlapping genes showing mitochondrial-related pathways among the top pathways potentially regulated by all three TFs. (E) Genome browser tracks of several mitochondrial-related genes showing localization of GLIS3, HNF1B, and NRF1 binding peaks within the same regulatory regions supporting the hypothesis that GLIS3 regulates these genes in coordination with HNF1B and NRF1. (For interpretation of the references to color in this figure legend, the reader is referred to the Web version of this article.)

GLIS3 regulates gene transcription by binding to a C-rich binding motif (GLISBS) in the regulatory regions of target genes [3,26]. To identify GLIS3-bound target genes, we performed ChIP-Seq analysis with kidneys from *Glis3*-EGFP mice. Comparison of the GLIS3 cistrome and transcriptome data revealed that of the 6793 genes up-regulated in WT kidneys during PND7-28, 46.5% bound GLIS3 (Figure 5A). Reactome pathway analysis of this gene set identified mitochondrial-related processes as the topmost significantly up-regulated pathways (Figure 5A). Analysis of our cistromics and transcriptomics data further showed that of the 5925 genes suppressed in *Glis3-*KO2 kidneys, 43.6% bound GLIS3 (Figure 5B). Reactome pathway analysis of this gene set also identified mitochondria-related processes as the top downregulated pathways, including the TCA cycle, mitochondrial translation, respiratory electron transport, and mitochondrial biogenesis-related processes (Figure 5B). These data suggest that in the postnatal kidney GLIS3 has a higher preference for binding to mitochondrial-related genes than genes of other pathways. Moreover, these observations are consistent with a direct role for GLIS3 in the transcriptional regulation of several mitochondrial function-related genes during kidney maturation.

HOMER analysis of the GLIS3 cistrome identified the consensus GLISBS among the top binding sites (Figure 5C). The genome browser tracks in Figure 5E show several examples of the localization of GLIS3 binding peaks within +/− 5 kb of the transcriptional start site of *Tfam*, *Tfb2m*, *Tfb1m, Mgme1, Ppargc1a*, *Ppargc1b, Sdha*, *Uqcrc2*, *Ndufs8*, and *Atp5j2*. GLIS3 binding peaks were not associated with any of the mitochondrial-encoded genes consistent with the concept that these genes are indirectly regulated by GLIS3. Interestingly, in addition to identifying GLISBS as a top consensus binding site, HOMER analysis of the GLIS3 cistrome revealed that binding sites for HNF1B and NRF1 frequently localized near GLIS3 binding loci (Figure 5C). Importantly, these TFs each have been implicated in the regulation of energy homeostasis and mitochondrial metabolism in several tissues, including the kidney [[27], [28], [29], [30], [31], [32], [33], [34], [35]]. Figure 5D shows that there was a significant overlap between the downregulated GLIS3 target genes and HNF1B- and NRF1-bound genes. Of the 2585 downregulated GLIS3 target genes, 1780 also bound HNF1B and NRF1, while 803 genes bound GLIS3 and HNF1B, but not NRF1. None of these genes bound both GLIS3 and NRF1, but not HNF1B. Reactome analysis of the 1780 GLIS3-, HNF1B-, and NRF1-bound genes again identified mitochondrial translation, TCA cycle, respiratory electron transport, and mitochondrial biogenesis-related processes among the top pathways (Figure 5D) as in the pathway analysis of all GLIS3-bound downregulated genes (Figure 5A). Supplementary Tables S1–4 provide a summary of the GLIS3/HNF1B/NRF1 binding peaks (5 kb within the TSS) associated with OXPHOS-, TCA cycle-, FAO-, and mitochondrial biogenesis-related genes that are downregulated in *Glis3-KO2* kidneys. The genome browser tracks in Figure 5E compares the locations of the binding peaks of endogenous GLIS3, HNF1B, and NRF1 near each other in *Tfam*, *Tfb2m*, *Tfb1m, Ppargc1a*, *Ppargc1b, Mgme1, Sdha*, *Uqcrc2*, *Ndufs8*, and *Atp5j2.* Genome browser tracks for several FAO-related genes (*Acad12, Acad10, Acadm, Etfa*) are shown in Supplementary Fig. S4. Together, these observations support the hypothesis that GLIS3 regulates the transcription of many key mitochondria-related genes in coordination with HNF1B and/or NRF1.

### 3.3 Mitochondrial DNA (mtDNA) and respiration are reduced in Glis3-KO2 kidneys

**Figure 6:**
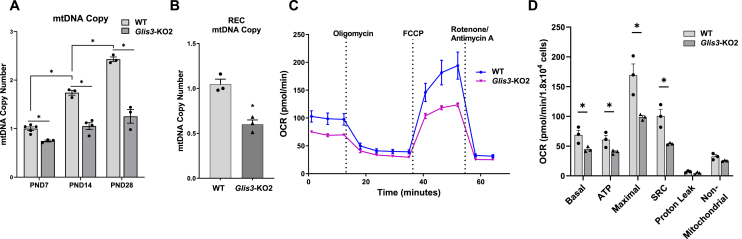
**Mitochondrial DNA and mitochondrial respiration are decreased in *Glis3-*KO2 kidneys.** (A) Analysis of mtDNA copy number in PND7, 14, and 28 WT and *Glis3-*KO2 kidneys. Data are represented as mean ± SEM, *n* ≥ 3. (B) mtDNA copy number in primary WT and *Glis3-*KO2 RECs. Data are represented as mean ± SEM, *n* = 3. (C) Oxygen consumption rate (OCR) was measured in primary RECs isolated from WT and *Glis3-*KO2 kidneys with a Seahorse analyzer. OCR was analyzed at the basal rate and after sequential injections of oligomycin (ATP synthase inhibitor), FCCP (proton uncoupler), and rotenone/antimycin A (Complex I and III inhibitor). Basal, ATP-linked (ATP), and maximal OCR, spare respiratory capacity (SCR), proton leak, and nonmitochondrial related OCR were then calculated and plotted. *n* ≥ 3. Primary RECS were isolated and cultured from individual mice and analyzed separately.

To relate the changes in gene expression to mitochondrial metabolism and function, we examined whether the downregulation of mitochondrial DNA replication/biogenesis-related genes in *Glis3-*KO2 kidneys (Figure 2D, Supplementary Table S3) resulted in any changes in mtDNA copy number and mitochondrial respiration. Analysis of mtDNA in WT and *Glis3-KO2* kidneys showed that mtDNA copy number steadily increased during the first month of kidney development in WT mice and that this increase was inhibited in *Glis3-*KO2 kidneys at all timepoints (Figure 6A). The reduction in mtDNA copy number in the *Glis3*-KO2 kidneys was consistent with the observed suppression of mitochondrial DNA-related transcription factors (Figure 3A). In addition, we observed a reduced mitochondria number and size in the cystic collecting duct epithelial cells whereas in the non-cystic collecting ducts only the mitochondria size was diminished (Supplementary Fig. S5). Likewise, mtDNA copy number was reduced in primary cultures of epithelial cells isolated from *Glis3*-KO2 mouse kidneys compared to that from WT kidneys (Figure 6B). The effect of loss of GLIS3 function on mitochondrial oxygen consumption rates (OCR) was analyzed in renal epithelial cells (RECs) with an XF96e Seahorse bioanalyzer [36]. The mitochondrial stress test demonstrated that basal respiration, ATP production, maximal respiration, and spare respiratory capacity (SRC) were all suppressed in *Glis3-*KO2 RECs compared to those in WT cells (Figure 6C,D), consistent with our transcriptome and pathway analysis (Figure 1, Figure 2; Figure 5B). Together, these data indicate that the observed decrease in the expression of mitochondria-related genes and proteins in *Glis3-*KO2 kidneys is at least in part responsible for the reduced mtDNA copy number and decreased utilization of mitochondrial respiration in *Glis3-*KO2 RECs.

### 3.4 Integrated multi-omic pathway analysis

**Figure 7:**
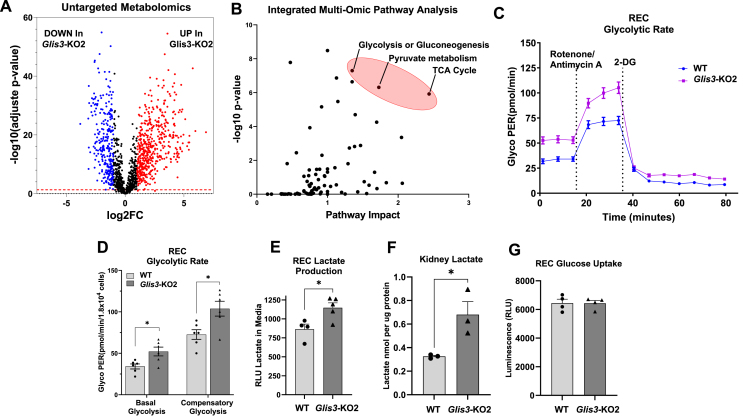
***Glis3-*KO2 kidneys have altered metabolites and an increase in aerobic glycolysis.** (A) Changes in metabolites identified by untargeted metabolomics. Volcano plot showing metabolites that are decreased (blue) or increased (red) in PND28 *Glis3-*KO2 kidneys. (B) Integrated multi-omic pathway analysis of RNA-seq DEGs and significantly changed metabolites identifying TCA cycle and glycolysis among the top altered pathways. (C) Glycolytic rate was measured in primary RECs isolated from WT and *Glis3-*KO2 kidneys with Seahorse analyzer after sequential injections of rotenone/antimycin A and 2-DG. (D) Basal and compensatory glycolysis were calculated and plotted. Primary RECS were isolated and cultured from three individual mice and analyzed separately. *n* = 3. (E) Analysis of lactate production in media from primary WT and *Glis3-*KO2 RECs. (F) Analysis of the lactate concentration in PND28 WT and *Glis3-*KO2 whole kidneys. (G) Analysis of the glucose uptake between primary WT and *Glis3-*KO2 RECs. Data are represented as mean ± SEM, *n* ≥ 3. (For interpretation of the references to color in this figure legend, the reader is referred to the Web version of this article.)

**Figure 8:**
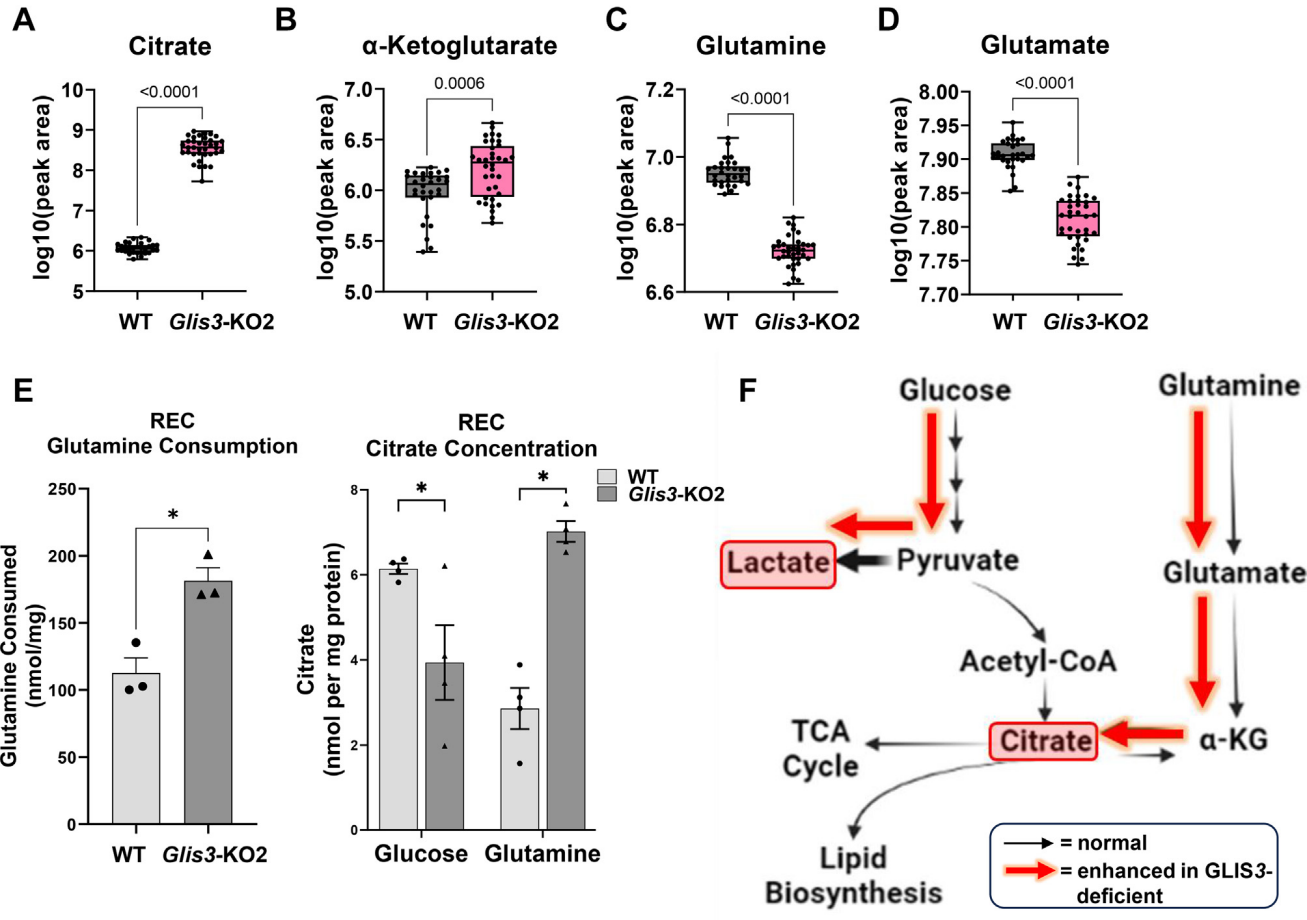
**Loss of GLIS3 function causes rewiring of glutamine and citrate metabolism in *Glis3-*KO2 kidneys.** (A–D) Untargeted metabolomic analysis of WT and *Glis3-*KO2 kidneys showed that citrate and α-ketoglutarate levels are increased, and glutamine and glutamate levels decreased (as log10, peak area). 5 biological replicates each with technical replicates. (E) Glutamine consumption is increased in the *Glis3-*KO2 RECs and citrate metabolism is altered in the primary *Glis3-*KO2 RECs. Glutamine consumption and citrate concentration measurements were analyzed as described in Methods. Data are represented as mean ± SEM, *n* ≥ 3. (F) Enhanced effect of GLIS3 deficiency in kidneys shown in red arrows on glucose and glutamine metabolic pathways. Loss of GLIS3 function in renal cells alters the utilization of glutamine and citrate levels consistent with enhanced glutamine anaplerosis. (For interpretation of the references to color in this figure legend, the reader is referred to the Web version of this article.)

An untargeted metabolomics approach was used to identify changes in metabolism in *Glis3-*KO2 kidneys associated with the suppression of mitochondrial-related gene expression and metabolic reprogramming. Metabolic analysis demonstrated that loss of GLIS3 function caused significant changes in the level of many metabolites (Figure 7A). Integrated multi-omic pathway (untargeted metabolomics and transcriptomics) analysis identified the TCA cycle, glycolysis, and pyruvate metabolism among the top impacted pathways (Figure 7B), like our gene profiling analysis (Figure 1, Figure 2). These changes in metabolism are consistent with the hypothesis that loss of GLIS3 function causes metabolic reprogramming in *Glis3-*KO2 kidneys. Analysis of glycolysis in RECs with the Seahorse glycolytic rate assay demonstrated that *Glis3-*KO2 RECs had a higher basal and compensatory glycolytic proton efflux rate (glycoPER) than WT cells indicating increased cellular glycolysis (Figure 7C,D). Higher lactate levels were observed in both *Glis3-*KO2 RECs and *Glis3*-KO2 kidneys compared to WT controls (Figure 7E,F) consistent with increased glycolysis and lower utilization of mitochondrial oxidative phosphorylation in *Glis3*-KO2 kidneys (Figure 6C,D); however, no difference was observed in glucose uptake between WT and *Glis3*-KO2 RECs (Figure 7G). PrestoBlue assay showed no significant differences in fluorescent signal between WT and *Glis3*-KO2 RECs indicating that the differences observed in the Seahorse assay were not due to differences in the number of metabolically active cells (not shown). Intriguingly, citrate and α-ketoglutarate, key TCA cycle metabolites, were found to be elevated in the *Glis3*-KO2 kidneys compared to WT in the untargeted metabolomics data (Figure 8A,B), despite transcriptomic and pathway analysis supporting a downregulation of the TCA cycle (Figure 1, Figure 5A). We examined glutamine and glutamate, as they are capable of contributing to the levels of α-ketoglutarate and citrate via glutamine anaplerosis or the glutamine reductive carboxylation pathway and found that both metabolites were lower in *Glis3-*KO2 kidneys (Figure 8C,D) [19,[37], [38], [39]]. Interestingly, *Glis3-*KO2 RECs had an increased glutamine consumption compared to WT (Figure 8E). Moreover, when fed glutamine only, *Glis3*-KO2 RECs increased the intracellular citrate concentration compared to WT indicating a potential contribution of glutamine to the high citrate levels observed in the *Glis3-*KO2 kidneys (Figure 8A,E). In comparison, when fed glucose only, *Glis3*-KO2 RECs had a decreased intracellular citrate concentration compared to WT (Figure 8E). Enhanced lactate production, increased glutamine consumption, and changes in TCA cycle metabolites, such as α-ketoglutarate and citrate, are important features of metabolic reprogramming (Figure 8F) [[40], [41], [42]]. These data further support GLIS3 being a critical regulator for proper physiological renal mitochondrial metabolism and that loss of GLIS3 function causes multiple metabolic perturbations.

### 3.5 Transcriptional regulation of mitochondria-related pathways in kidney-selective knockout (Glis3-Pax8Cre) mice mimic that of ubiquitous GLIS3-deficient mice

**Figure 9:**
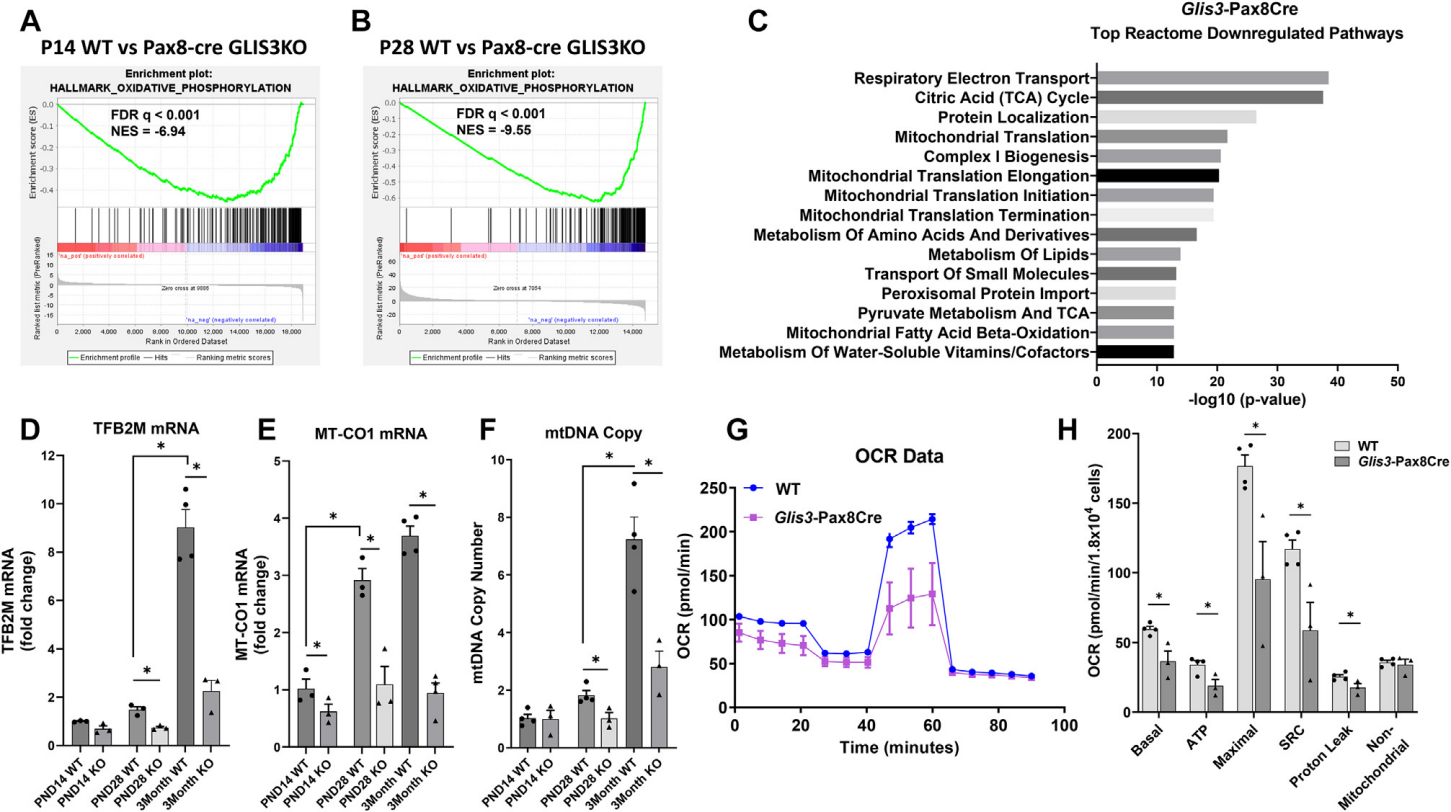
**Kidney-selective knockout *Glis3*-Pax8Cre mice mimic the ubiquitous *Glis3-*KO2 renal metabolic reprogramming features.** (A–B) Enrichment plots of the Hallmark OXPHOS gene set showing negative enrichment in *Glis3*-Pax8Cre kidneys at PND14 and PND28. (C) Reactome pathway analysis of downregulated genes in *Glis3*-Pax8Cre kidneys showed that mitochondrial-related pathways are among the top downregulated pathways. (D–E) RT-qPCR analysis of the expression of *Tfb2m* and *mt–CO1* genes in WT and *Glis3*-Pax8Cre kidneys at the ages indicated. Data are represented as mean ± SEM, *n* ≥ 4. (F) Comparison of mtDNA copy number between kidneys from PND14, 28, and 3-month WT and *Glis3-*Pax8Cre mice. Data are represented as mean ± SEM, *n* ≥ 3. (G–H) Profile of mitochondria stress test of primary RECs isolated from WT and *Glis3*-Pax8Cre kidneys. OCR was analyzed with a Seahorse analyzer. Basal, ATP-linked, and maximal OCR, SCR, proton leak, and nonmitochondrial related OCR were calculated and plotted. *n* ≥ 3. Primary RECS were isolated and cultured from individual mice and analyzed separately.

In addition to PKD, ubiquitous GLIS3-deficient mice develop neonatal diabetes and hyperglycemia [[4], [5], [6]], which are well-established risk factors in the development of renal dysfunction and diabetic nephropathy [38]. Therefore, to exclude neonatal diabetes and hyperglycemia as variables, we analyzed mitochondrial and metabolic gene expression and functions in kidney-selective GLIS3-deficient (*Glis3*-Pax8Cre) mice that do not develop neonatal diabetes. Analysis of genes downregulated in *Glis3*-Pax8Cre kidneys confirmed the observations made with ubiquitous *Glis3*-KO2 kidneys (Figure 1C). GSEA analysis identified OXPHOS as the top negatively enriched pathway at PND14 and PND28 (Figure 9A,B), while Reactome pathway analysis identified respiratory electron transport, TCA cycle, mitochondrial translation, amino acid metabolic pathways, and lipid metabolism among the top downregulated pathways in PND28 *Glis3*-Pax8Cre kidneys (Figure 9C). Heatmap analysis of the MitoCarta gene set of the WT and *Glis3*-Pax8Cre kidneys (Supplementary Fig. S6) showed a very similar pattern of induction during PND7-28 in WT kidneys. It further demonstrated that most genes were greatly suppressed in PND28 *Glis3*-Pax8Cre kidneys, but not completely blocked very similar to the gene expression profile observed in *Glis3*-KO2 kidneys (Figure 2C). The expression of *Tfb2m* and *mt–Co1* RNA, as well as mtDNA copy number, were reduced as observed in ubiquitous *Glis3*-KO2 kidneys (Figure 9D,F). Furthermore, the Seahorse mito-stress test showed that primary *Glis3*-Pax8Cre RECS exhibited an overall lower mitochondrial OCR compared to WT cells (Figure 9G,H) as ubiquitous *Glis3*-KO2 RECs (Figure 6C,D). These data indicate that the transcriptomic and phenotypic changes observed in *Glis3*-Pax8Cre kidneys mimic those observed in ubiquitous *Glis3*-KO2 kidneys suggesting that these changes are mainly due to the absence of GLIS3 expression in the kidney rather than neonatal diabetes and hyperglycemia.

## 4 Discussion

Previously, we and others reported that loss of GLIS3 function in humans and mice causes autosomal recessive PKD [2,[4], [5], [6]]. Changes in cellular metabolism and energy homeostasis are critical components of normal postnatal kidney development and are also important in PKD pathogenesis [13,[18], [19], [20],^43^]. To examine the potential role of GLIS3 in the regulation of energy homeostasis and mitochondria functions in normal kidney and PKD pathogenesis, we performed transcriptome and cistrome analysis with kidneys from WT and *Glis3*-KO2 mice at different times during early postnatal kidney maturation. Transcriptome analysis showed that in WT kidneys the expression of many genes associated with mitochondria-related functions, such as the respiratory electron transport chain, TCA cycle, and mitochondrial biogenesis, are steadily enhanced during the first postnatal month, consistent with previous studies showing an increased reliance on energy generated by OXPHOS rather than aerobic glycolysis during normal renal maturation (Figure 1A–C) [23,25,44]. In contrast, in *Glis3-*KO2 kidneys, respiratory electron transport chain, TCA cycle, and mitochondrial biogenesis genes are increasingly suppressed (Figure 1, Figure 2D,E). These data indicated that the normal shift from aerobic glycolysis to oxidative phosphorylation during PND7-28 is significantly repressed in *Glis3-*KO2 kidneys. This metabolic reprogramming is supported by the observed decrease in mtDNA copy number in *Glis3-KO2* kidneys and Seahorse analysis showing reduced mitochondrial OCRs, a higher basal and compensatory glycolytic proton efflux rate (glycoPER), altered glutamine utilization, and increased lactate production in *Glis3-*KO2 RECs (Figure 6A–D; Figure 7C–F). Together, these findings identify GLIS3 as a new participant in the regulation of the metabolic transition from aerobic glycolysis to OXPHOS during early postnatal kidney development by promoting the expression of OXPHOS- and mitochondrial biogenesis-related genes. Energy generated by aerobic glycolysis rather than from the utilization of mitochondrial OXPHOS is often associated with highly proliferating cells, including various cancer cells, and is believed to promote cell proliferation and cyst formation [13,18,[45], [46], [47], [48], [49]]. Although these features of metabolic reprogramming observed in *Glis3-*deficient kidneys may contribute to renal cystogenesis, further studies are needed to determine the extent to which it contributes to the initiation and progression of renal cyst formation.

Because GLIS3 functions as a transcription factor, we hypothesized that the transcription of several metabolic and mitochondrial genes during early postnatal kidney development might be regulated directly by GLIS3. We previously reported that GLIS3 regulates gene transcription in the thyroid and pancreatic beta cells by binding to GC-rich binding sequences (GLISBS) in regulatory regions of target genes, such as *Slc5a5* in thyroid follicular cells and *Ins2* in beta cells [2,3]. Cistrome analysis of mouse kidneys revealed that GLIS3 binding peaks are localized within many genes with roles in the TCA cycle, respiratory electron transport chain, and mitochondrial biogenesis, the majority of which are suppressed in *Glis3-*KO2 kidneys (Figure 5A and Supplementary Tables S1–4). These observations indicate that GLIS3 functions as a transcriptional activator by binding to GLISBS in regulatory regions within these genes. Since GLIS3 was not found to regulate mitochondrial functions in several other tissues (Supplementary Fig. S3) [2,3], the transcriptional regulation of mitochondrial biogenesis, TCA cycle, and OXPHOS by GLIS3 may be relatively kidney selective and particularly important during the first month of postnatal renal maturation and in cystogenesis. Interestingly, a recent study reported that *GLIS3* expression was decreased in an early-onset autosomal dominant polycystic kidney disease (ADPKD) model [50], consistent with the concept that down-regulation of GLIS3 may play a role in ADPKD development and/or progression similar to *Glis3*-deficiency, thereby increasing the significance of our findings to the study of renal cystic disease.

**Figure 10:**
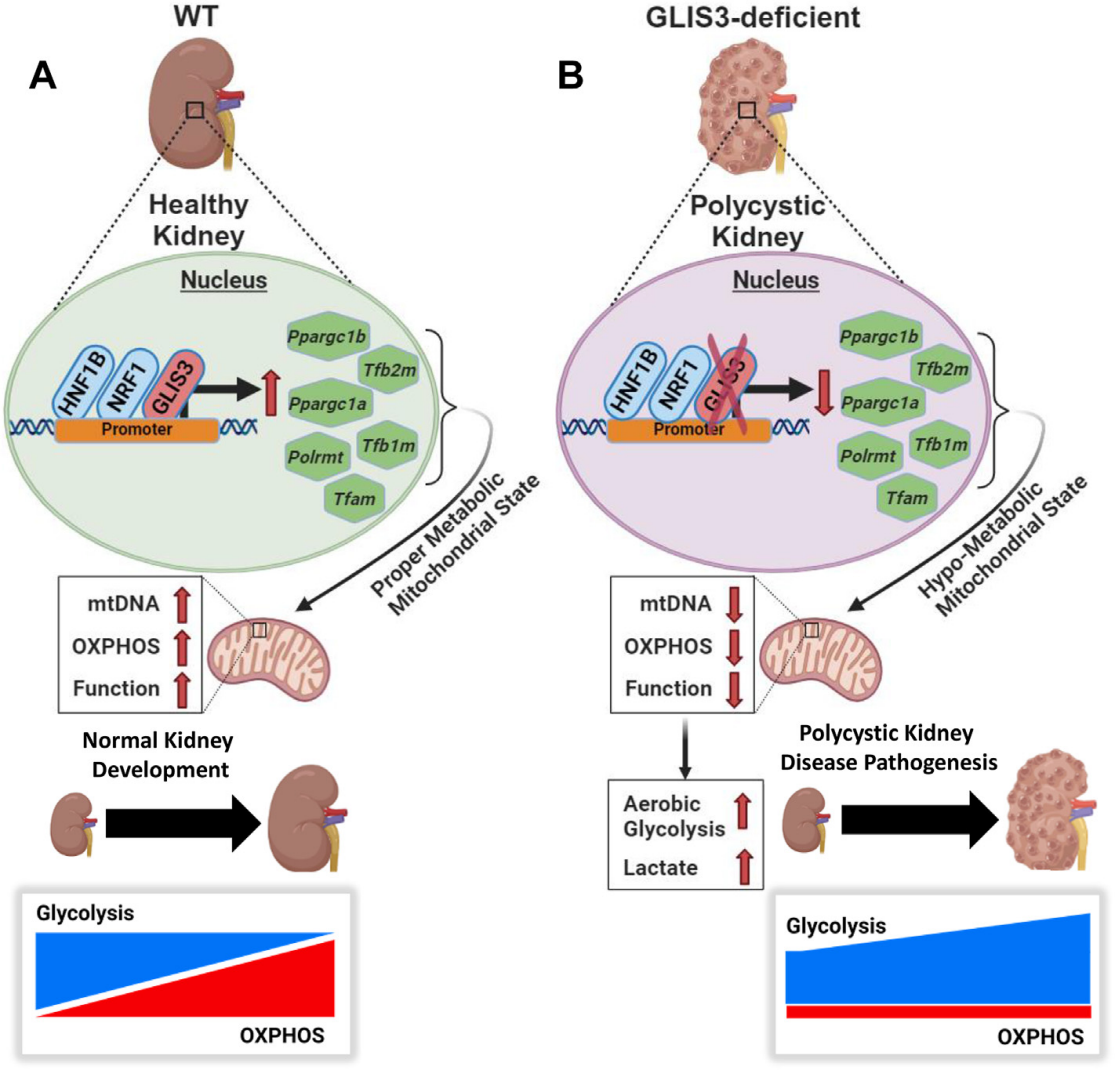
**Schematic of the transcriptional regulation of mitochondrial and metabolic genes by GLIS3 likely in coordination with HNF1B and NRF1.** (A) GLIS3 activates the transcription of several mitochondrial and metabolic genes during postnatal kidney development in coordination with HNF1B and NRF1. However, some genes are likely regulated indirectly via the increased expression of other key regulators of transcription (e.g., *Tfam, Tfb1/2m, Ppargc1a/b, Polrmt*) that themselves are GLIS3 transcriptional targets. Together this enhances mitochondrial-related gene expression and OXPHOS. (B) Loss of GLIS3 function suppresses the normal induction of these mitochondrial-related genes leading to a decrease in mtDNA and OXPHOS compared to WT, and an increase in aerobic glycolysis and lactate production.

Comprehensive HOMER analysis of our GLIS3 ChIP-Seq data identified GLISBS among the top binding sites and further revealed that binding sites for HNF1B and NRF1 are frequently localized near GLIS3 binding peaks. This suggests that GLIS3 may regulate the transcription of many of these genes in coordination with these TFs, however direct interaction with these TFs have not been demonstrated. This conclusion was supported by the collective analysis of the *Glis3*-KO2 transcriptome and the GLIS3, HNF1B, and NRF1 cistromes showing significant overlaps between the binding of GLIS3, HNF1B, and/or NRF1 to genes downregulated in *Glis3-*KO2 kidneys (Figure 5A,D). Reactome pathway analysis of downregulated genes with binding peaks for all three TFs identified various mitochondria-related processes among the top pathways and included several mitochondrial biogenesis-, OXPHOS-, FAO, and TCA cycle-related genes (Supplementary Tables S1–4) and several mitochondrial-function-regulatory genes (e.g., *Tfam*, *Tfb2m*, *Tfb1m*, and *Ppargc1a/b*) (Figure 5D,E) [51]. These observations are consistent with studies showing a role for HNF1B in the regulation of mitochondrial metabolism in ovarian cancers and in renal cells [32,52], and with the role of NRFs in the regulation of mitochondrial biogenesis and the transcriptional regulation of *Tfb2m*, *Tfam,* and *Tfb1m* (Figure 5, Figure 10) [28,29,[53], [54], [55]].

Expression of mitochondrial function-related genes and energy homeostasis are under complex transcriptional and posttranscriptional controls [54,55]. Transcription of mitochondrial genes is regulated by single promoters on each strand generating two polycistronic transcripts [56,57]. TFAM, POLRMT, and TFB2M are part of a mitochondrial transcription complex required for transcription of mitochondrial genes and mitochondrial biogenesis. TFAM regulates mitochondrial gene transcription by recruiting POLRMT to specific promoters, while TFB2M induces structural changes in POLRMT [[56], [57], [58], [59], [60]]. Because mitochondria-encoded genes are not directly regulated by GLIS3 and *Tfam*, *Polrmt*, and *Tfb2m* mRNA expression are suppressed in *Glis3*-KO kidneys, the downregulation of these genes may be in part responsible for the suppression of mitochondria-encoded genes and the reduction in mtDNA copy number observed in GLIS3-deficient kidneys, potentially contributing to cystogenesis. The latter is consistent with a study showing that TFAM-deficiency results in progressive mitochondrial depletion and severe cystic kidney disease [59]. The reduced expression of *Ppargc1a* and *Ppargc1b,* which encode the transcriptional coactivators PGC-1α and PGC-1β, respectively, was also of interest since these proteins play a critical role in the regulation of mitochondrial functions (biogenesis, energy homeostasis, and oxidative stress) [54,55]. PGC-1 family of coactivators interact with numerous transcription factors, including several nuclear receptors (e.g., peroxisome proliferator receptors; PPARs), NRF1/2, and TFAM [31,54,61,62]. NRF1 promotes mitochondrial biogenesis alongside NRF2 and PGC-1α [31]. Binding peaks for GLIS3 and HNF1B localize within the same region of *Ppargc1a*, while GLIS3, HNF1B, and NRF1 bind near each other within *Ppargc1b* (Figure 5E) [8,32]. The decreased *Ppargc1a/b* expression likely decreases the transcriptional activity of NRFs, PPARs, and TFAM resulting in reduced expression of their downstream target genes, including mitochondrial biogenesis-, OXPHOS-, TCA cycle-, and FAO-related genes (Figure 10). Thus, GLIS3 regulation of *Ppargc1a/b* expression in the early postnatal kidney might be part of the mechanism underlying metabolic reprogramming and the decrease in mtDNA copy number observed in GLIS3-deficient kidneys. Further studies are needed to obtain greater insights into the relationship between GLIS3 and other transcriptional regulators in the control of mitochondrial-related gene transcription. In this study, we focused on ubiquitous Glis3 knockout mice; however, cystogenesis caused by loss of GLIS3 function might be dependent on the organ’s developmental status as reported for *PKD1* [63]. Study of an inducible GLIS3 knockout model could help to further clarify the role of GLIS3 in kidney metabolic regulation and cyst formation beyond early postnatal timepoints.

## 5 Conclusions

In this study, we identify the transcription factor GLIS3 as a new participant in an interconnected transcription regulatory network that regulates the metabolic transition from aerobic glycolysis to OXPHOS during the first month of normal postnatal kidney development (Figure 10). We provide evidence indicating that GLIS3, in coordination with HNF1B and NRF1, directly regulates the transcription of many metabolic and mitochondrial function-related genes and that it indirectly regulates mitochondria encoded genes via its transcriptional regulation of *Tfam*, *Tfb2m*, *Polrmt*, and *Ppargc1a/b*. Loss of GLIS3 function suppresses the expression of these metabolic genes and promotes features of metabolic reprogramming resulting in decreased OXPHOS and increased aerobic glycolysis. Our study suggests that GLIS3 may have a protective role in the postnatal kidney against cyst formation and that targeting GLIS3 signaling may offer therapeutic opportunities in the management of polycystic kidney disease.

## CRediT authorship contribution statement

**Justin B. Collier:** Writing – review & editing, Writing – original draft, Visualization, Supervision, Investigation, Formal analysis, Conceptualization. **Hong Soon Kang:** Writing – review & editing, Methodology, Investigation. **Yun-Gil Roh:** Investigation. **Chitrangda Srivastava:** Investigation. **Sara A. Grimm:** Writing – review & editing, Formal analysis, Data curation. **Alan K. Jarmusch:** Writing – review & editing, Formal analysis, Data curation. **Anton M. Jetten:** Writing – review & editing, Visualization, Supervision, Conceptualization.

## Funding

This research was supported by the Intramural Research Program of the 10.13039/100000066NIEHS, NIH Z01-ES-101585.

## Declaration of competing interest

All the authors declared no competing interests.

## Data Availability

Data will be made available on request.
