# Supplementary material for: GLIS3: A novel transcriptional regulator of mitochondrial functions and metabolic reprogramming in postnatal kidney and polycystic kidney disease
Source: Mol Metab. 2024 Nov 5;90:102052. doi: 10.1016/j.molmet.2024.102052 (PMC11613186; doi:10.1016/j.molmet.2024.102052)
Supplement: Multimedia component 1 [file mmc1.docx]

Supplementary Materials

**GLIS3: a novel transcriptional regulator of mitochondrial functions and metabolic reprogramming in postnatal kidney and polycystic kidney disease**

Justin B. Collier^1^, Hong Soon Kang^1^, Yun-Gil Roh^1^, Chitrangda Srivastava^1^,

Sara A. Grimm^2^, Alan K. Jarmusch^3^, and Anton M. Jetten^1^*

^1^Cell Biology Group, Immunity, Inflammation and Disease Laboratory

^2^Integrative Bioinformatics

^3^Metabolomics Core Facility, Immunity, Inflammation and Disease Laboratory

National Institute of Environmental Health Sciences

National Institutes of Health

Research Triangle Park, NC 27709, USA

^*^ To whom correspondence should be addressed

### E-mail: jetten@niehs.nih.gov

**Table of Contents**

**Supplementary Figures**

Supplementary Figure S1. Gene ontology cellular component analysis shows genes related to mitochondrial compartments are suppressed in *Glis3*-KO2 kidneys. (page 4)

Supplementary Figure S2. Progression of renal cystogenesis in *Glis3*-KO2 mice during the first postnatal month. (page 5)

Supplementary Figure S3. Loss of GLIS3 function affects *Tfb2m* and *Tfam* mRNA expression in a tissue selective manner. (page 6)

Supplementary Figure S4. Genome browser tracks of several mitochondrial FAO-related genes showing localization of GLIS3, HNF1B, and NRF1 binding peaks within the same regulatory regions. (page 7)

Supplementary Figure S5. Mitochondria number and size are reduced in Glis3-KO2 cystic collecting ducts. (page 8)

Supplementary Figure S6. Heat Map showing the expression of mitochondria-related genes is decreased in *Glis3*-Pax8Cre kidneys. (page 9)

**Supplementary Tables**

Supplementary Table S1. List of OXPHOS-related genes that are differentially expressed in kidneys from *Glis3-KO2* and/or *Glis3*-Pax8Cre PND28 mice and contain a GLIS3, HNF1B, and/or NRF1 binding peak (indicated by +) within +/-5kb of the corresponding TSS. (page 10)

Supplementary Table S2. List of TCA cycle related genes that are differentially expressed in kidneys from *Glis3*-KO2 and/or *Glis3*-Pax8Cre PND28 mice and contain a GLIS3, HNF1B, and/or NRF1 binding peak (indicated by +) within +/-5kb from the corresponding TSS. (page 14)

Supplementary Table S3. List of mitochondrial biogenesis-related genes that are differentially expressed in kidneys from *Glis3*-KO2 and/or *Glis3*-Pax8Cre PND28 mice and contain a GLIS3, HNF1B, and/or NRF1 binding peak (indicated by +) within +/-5kb from the corresponding TSS. (page 15)

Supplementary Table S4. List of fatty acid oxidation-related genes that are differentially expressed in kidneys from *Glis3*-KO2 and/or *Glis3*-Pax8Cre PND28 mice and contain a GLIS3, HNF1B, and/or NRF1 binding peak (indicated by +) within +/-5kb from the corresponding TSS. (page 18)

Supplementary Table S5. Primers for QPCR analysis. (page 21)

**Supplementary Methods (page 22)**

**Reference (page 31)**

**
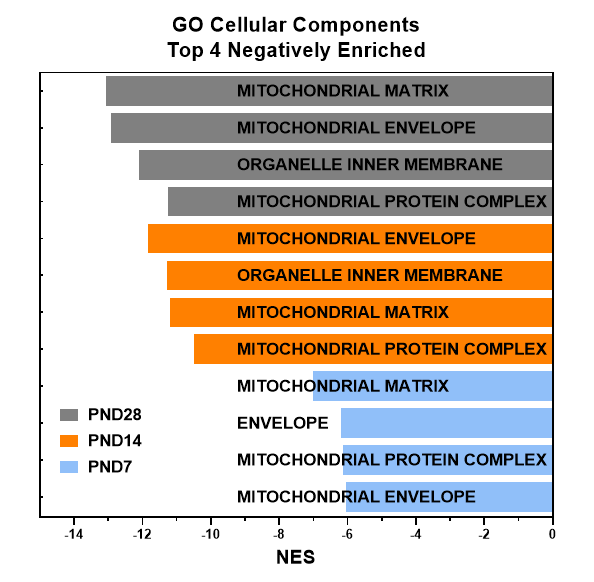
**

**Supplementary Figure S1.** **Gene ontology cellular component analysis shows genes related to mitochondrial compartments are suppressed in *Glis3*-KO2 kidneys.** Top GSEA negatively enriched Gene Ontology cellular component analysis in *Glis3-KO2* kidneys at PND7, 14, and 28 ranked by NES (normalized enrichment score), showing considerable mitochondria involvement.


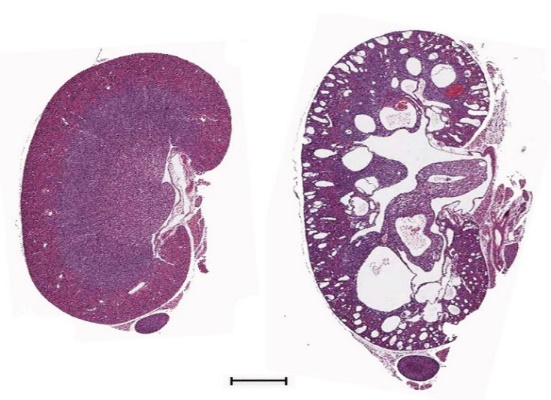

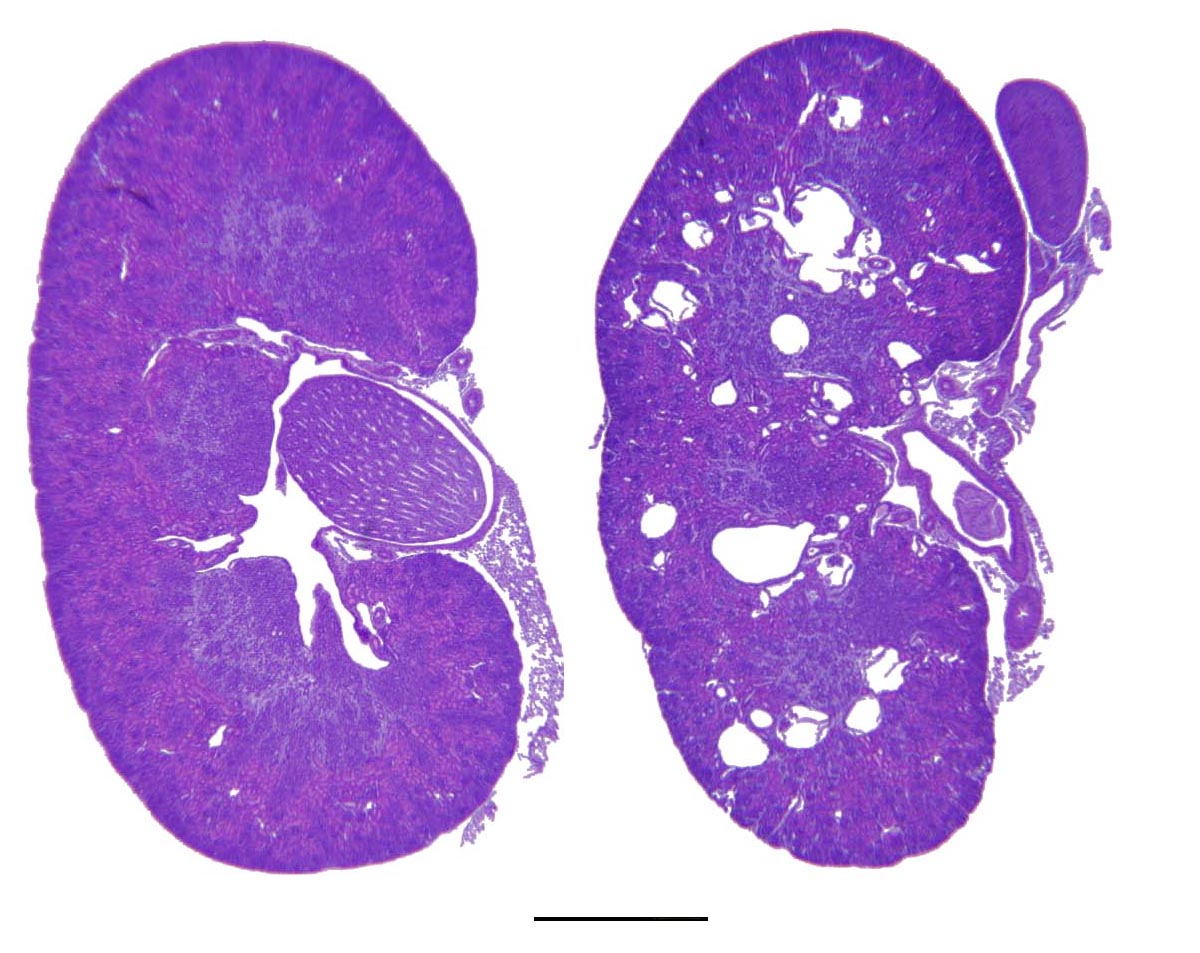

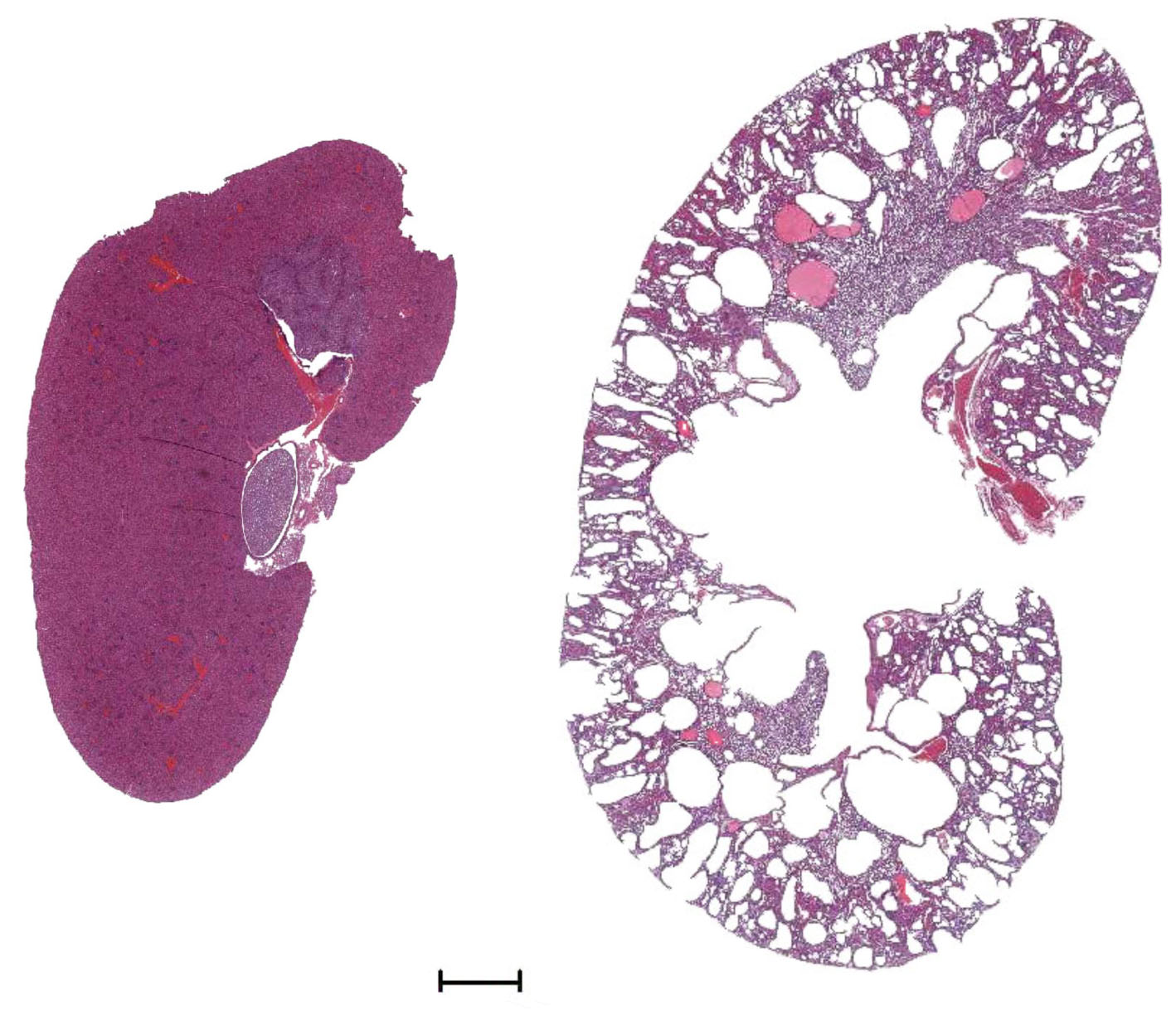


**PND7**

**PND28**

**PND14**

**WT**

***Glis3-KO2***

**Supplementary Figure S2.** **Progression of renal cystogenesis in *Glis3*-KO2 mice during the first postnatal month.** WT and *Glis3*-KO2 kidneys were stained with hematoxylin/eosin at PND7, PND14, and PND28. Representative sections are shown.


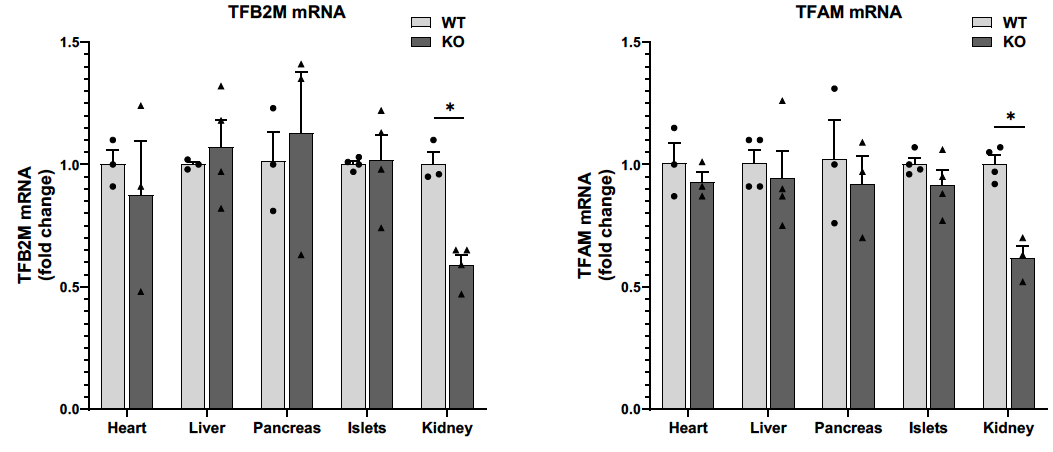


**Supplementary Figure S3 (associated with Fig. 3).** **Loss of GLIS3 function affects *Tfb2m* and *Tfam* mRNA expression in a tissue selective manner.** *Tfb2m* and *Tfam* mRNA expression was examined by RT-qPCR in the tissues indicated. Data are represented as mean ± SEM, kidney and islets *n* ≥ 4; pancreas, liver, and heart *n* ≥ 3, kidney and islets *n* ≥ 4; pancreas, heart, liver *n* ≥ 3.

**
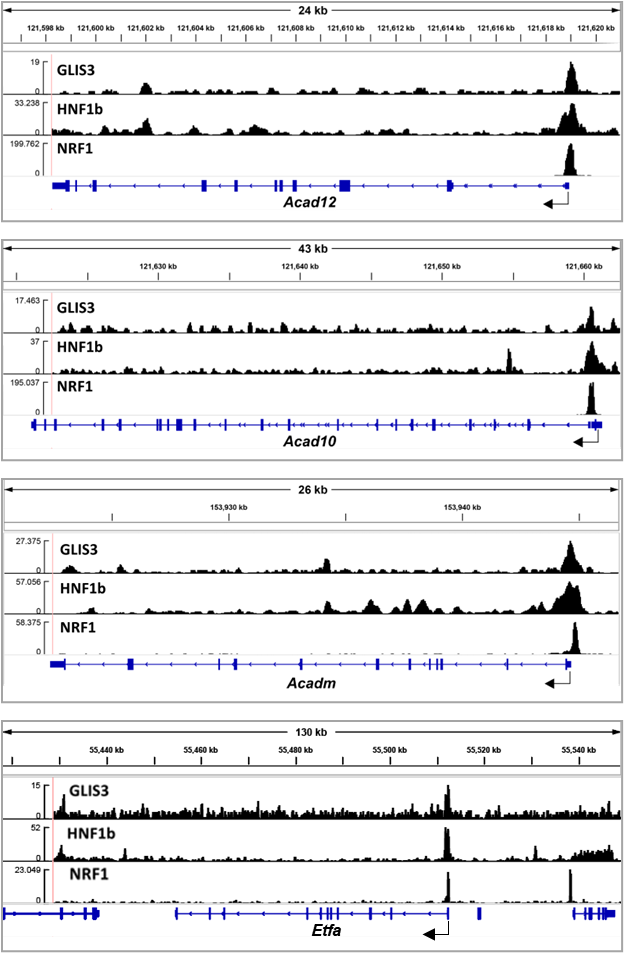
**

**Supplementary Figure S4 (associated with Fig. 5).** **Genome browser tracks of several mitochondrial FAO-related genes showing localization of GLIS3, HNF1B, and NRF1 binding peaks within the same regulatory regions.**


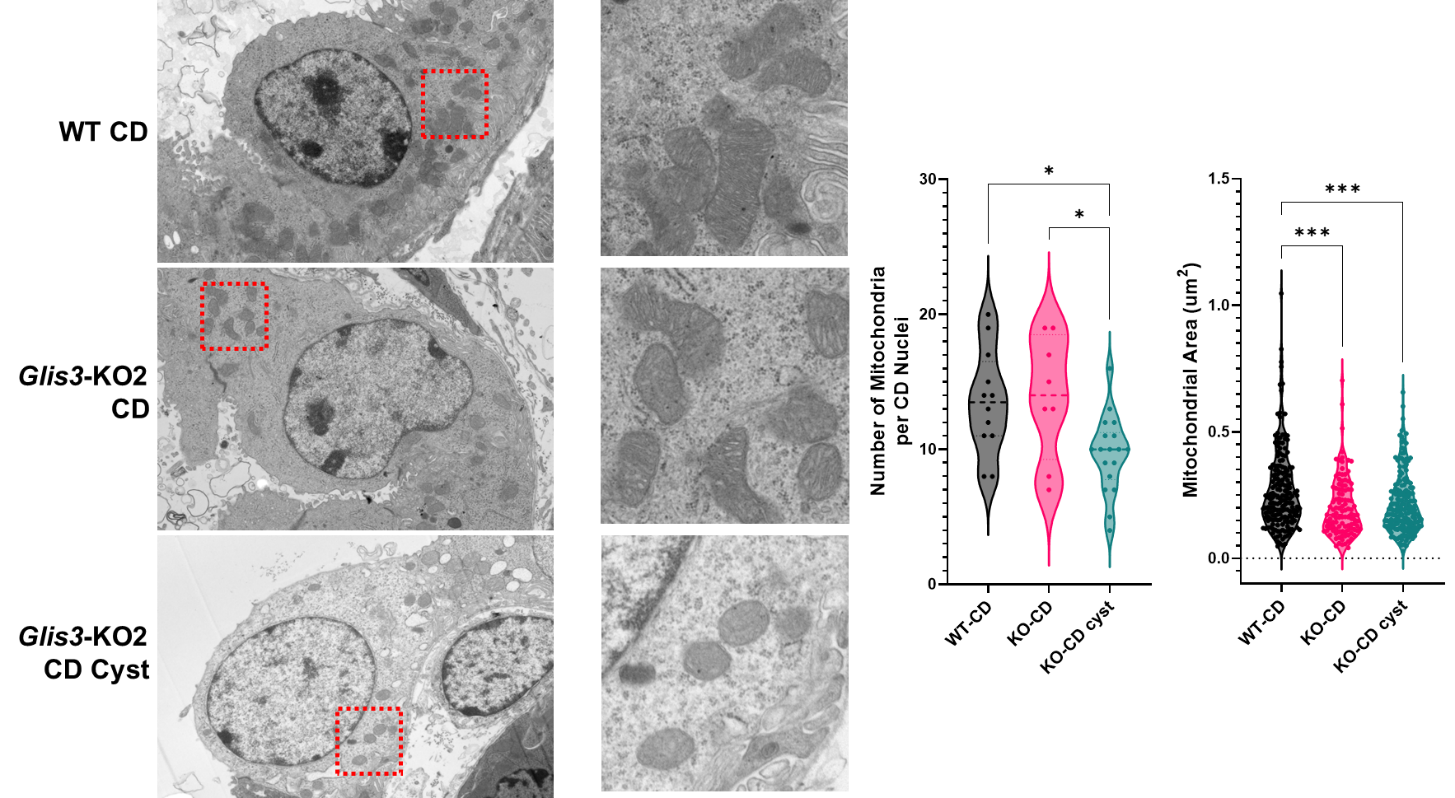


**Supplementary Figure S5.** **Mitochondria number and size are reduced in *Glis3*-KO2 cystic collecting ducts.** Three WT and three *Glis3*-KO2 kidney samples were processed for transmission electron microscopy (TEM) for comparing health and morphology of mitochondria. TEM images (3000x and 10000x) displaying representative collecting duct structures from WT and *Glis3*-KO2 kidneys from PND14. A total of 86 images from 11 different areas was analyzed for the WT samples and 117 images from 11 different areas was analyzed for the *Glis3*-KO2 samples. Volcano plot data show the number of mitochondria per collecting duct cell and mitochondria area (µm^2^). *** represents p < 0.001; * represents p < 0.05.

***Glis3-Pax8cre***

**Supplementary Figure S6 (associated with Fig. 9).** Heatmap of the MitoCarta 3.0 gene set in alphabetical order. Gene expression is compared between PND7 and PND28 WT and *Glis3-*KO2 kidneys. Upregulated genes are represented in red and downregulated genes in blue. Expression values are shown as z-scores of the rlog-transformed values for each gene.

**Supplementary Table S1.** **List of OXPHOS-related genes that are differentially expressed in kidneys from *Glis3-*KO2 and/or *Glis3*-Pax8Cre PND28 mice and contain a GLIS3, HNF1B, and/or NRF1 binding peak (indicated by +) within +/-5kb from the corresponding TSS.**

| **OXPHOS Genes** | | | | | |
| --- | --- | --- | --- | --- | --- |
| **gene symbol** | **PND28 *Glis3*-KO2** | **PND28 Pax8-Cre *Glis3*-KO2** | **GLIS3 Binding** | **HNF1B Binding** | **NRF1 Binding** |
| Atp4a | down | down |  |  |  |
| Atp5a1 | down | down |  |  | + |
| Atp5b | down | down |  |  | + |
| Atp5c1 | down | down | + | + | + |
| Atp5d | down | down | + | + | + |
| Atp5e | down | unchanged |  |  | + |
| Atp5f1 | down | down | + | + | + |
| Atp5g1 | down | down |  |  | + |
| Atp5g2 | down | down | + | + | + |
| Atp5g3 | down | down | + | + | + |
| Atp5h | down | down |  |  | + |
| Atp5j | down | down | + | + | + |
| Atp5j2 | down | down | + | + | + |
| Atp5k | down | down | + | + | + |
| Atp5l | down | down | + | + | + |
| Atp5o | down | down | + | + | + |
| Atp6ap1 | down | down |  |  | + |
| Atp6v0a1 | up | unchanged | + | + | + |
| Atp6v0a2 | down | down |  |  | + |
| Atp6v0a4 | down | down | + | + |  |
| Atp6v0b | down | unchanged |  |  | + |
| Atp6v0c | down | down |  |  | + |
| Atp6v0d1 | down | down | + | + | + |
| Atp6v0d2 | up | down |  |  |  |
| Atp6v0e | unchanged | down | + | + | + |
| Atp6v0e2 | down | down |  |  |  |
| Atp6v1a | down | down | + | + | + |
| Atp6v1b1 | down | down | + | + |  |
| Atp6v1b2 | down | down | + | + | + |
| Atp6v1c1 | down | down | + | + | + |
| Atp6v1c2 | down | down |  |  | + |
| Atp6v1d | down | down | + | + | + |
| Atp6v1e1 | down | down | + | + | + |
| Atp6v1f | down | down | + | + | + |
| Atp6v1g1 | down | down |  |  | + |
| Atp6v1g2 | up | unchanged |  |  | + |
| Atp6v1g3 | down | down |  |  |  |
| Atp6v1h | down | down | + | + | + |
| Cox10 | down | down |  |  | + |
| Cox11 | down | down | + | + |  |
| Cox15 | down | down | + | + | + |
| Cox17 | down | down | + | + | + |
| Cox4i1 | down | down |  |  |  |
| Cox5a | down | down | + | + | + |
| Cox5b | down | down | + | + | + |
| Cox6a1 | down | down | + | + | + |
| Cox6a2 | down | down |  |  |  |
| Cox6b1 | down | down | + | + |  |
| Cox6b2 | down | down |  |  |  |
| Cox6c | down | down |  |  | + |
| Cox7a1 | down | unchanged |  |  |  |
| Cox7a2 | down | down | + | + | + |
| Cox7a2l | unchanged | down |  |  | + |
| Cox7b | down | down |  |  | + |
| Cox7c | down | down |  |  | + |
| Cox8a | down | down | + | + | + |
| Cyc1 | down | down | + | + | + |
| Lhpp | up | unchanged | + | + | + |
| mt-Atp6 | unchanged | down |  |  |  |
| mt-Co1 | down | down |  |  |  |
| mt-Co2 | unchanged | down |  |  |  |
| mt-Co3 | unchanged | down |  |  |  |
| mt-Cytb | down | down |  |  |  |
| mt-Nd1 | down | down |  |  |  |
| mt-Nd2 | down | down |  |  |  |
| mt-Nd4 | down | down |  |  |  |
| mt-Nd4l | unchanged | down |  |  |  |
| mt-Nd5 | down | down |  |  |  |
| mt-Nd6 | down | down |  |  |  |
| Ndufa1 | down | down |  |  |  |
| Ndufa10 | down | down | + | + | + |
| Ndufa11 | down | down |  |  | + |
| Ndufa2 | down | down |  |  | + |
| Ndufa3 | down | down |  |  | + |
| Ndufa4 | down | down | + | + |  |
| Ndufa4l2 | unchanged | unchanged |  |  | + |
| Ndufa5 | down | down | + | + | + |
| Ndufa6 | down | down |  |  | + |
| Ndufa7 | down | unchanged |  |  | + |
| Ndufa8 | down | down |  |  | + |
| Ndufa9 | down | down | + | + | + |
| Ndufab1 | down | down | + | + | + |
| Ndufb10 | down | down |  |  | + |
| Ndufb2 | down | down |  |  | + |
| Ndufb3 | down | down | + | + |  |
| Ndufb4 | down | unchanged |  |  | + |
| Ndufb5 | down | down | + | + | + |
| Ndufb6 | down | down | + | + | + |
| Ndufb7 | down | down | + | + | + |
| Ndufb8 | down | unchanged |  |  | + |
| Ndufb9 | down | down |  |  | + |
| Ndufc1 | down | down |  |  | + |
| Ndufc2 | down | down |  |  | + |
| Ndufs1 | down | down | + | + | + |
| Ndufs2 | down | down | + | + | + |
| Ndufs3 | down | down |  |  | + |
| Ndufs4 | down | down |  |  | + |
| Ndufs5 | down | down | + | + | + |
| Ndufs6 | down | down | + | + | + |
| Ndufs7 | down | down |  |  | + |
| Ndufs8 | down | down | + | + | + |
| Ndufv1 | down | down | + | + | + |
| Ndufv2 | down | down | + | + | + |
| Ndufv3 | down | down |  |  | + |
| Ppa1 | down | unchanged |  |  | + |
| Ppa2 | down | down |  |  | + |
| Sdha | down | down | + | + | + |
| Sdhb | down | down | + | + | + |
| Sdhc | down | down | + | + |  |
| Sdhd | down | down | + | + | + |
| Tcirg1 | unchanged | up |  |  |  |
| Uqcr10 | down | down |  |  | + |
| Uqcr11 | down | down |  |  | + |
| Uqcrb | down | down |  |  | + |
| Uqcrc1 | down | down |  |  | + |
| Uqcrc2 | down | down | + | + |  |
| Uqcrfs1 | down | down | + | + | + |
| Uqcrh | down | unchanged |  |  | + |
| Uqcrq | down | down | + | + |  |

**Supplementary Table S2: List of TCA cycle related genes that are differentially expressed in kidneys from *Glis3-*KO2 and/or *Glis3*-Pax8Cre PND28 mice and contain a GLIS3, HNF1B, and/or NRF1 binding peak (indicated by +) within +/-5kb from the corresponding TSS.**

| **TCA Cycle Genes** | | | | | |
| --- | --- | --- | --- | --- | --- |
| **gene symbol** | **PND28 *Glis3*-KO2** | **PND28 Pax8-Cre *Glis3*-KO2** | **GLIS3 Binding** | **HNF1B Binding** | **NRF1 Binding** |
| Acly | unchanged | unchanged | + | + | + |
| Aco1 | down | down | + | + |  |
| Aco2 | down | down | + | + | + |
| Cs | down | down | + | + | + |
| Dlat | down | down | + | + | + |
| Dld | down | down | + |  | + |
| Dlst | down | down |  | + | + |
| Fh1 | down | down | + |  | + |
| Idh1 | down | unchanged | + | + |  |
| Idh2 | down | down | + | + |  |
| Idh3a | down | down | + |  | + |
| Idh3b | down | down |  | + | + |
| Idh3g | down | down |  | + | + |
| Mdh1 | down | down | + | + | + |
| Mdh2 | down | down | + | + | + |
| Ogdh | down | down |  | + | + |
| Ogdhl | down | down | + | + | + |
| Pck1 | down | down | + | + |  |
| Pck2 | up | unchanged | + |  | + |
| Pcx | down | down |  |  | + |
| Pdha1 | down | down | + | + | + |
| Pdhb | down | down | + | + | + |
| Sdha | down | down | + | + | + |
| Sdhb | down | down | + | + | + |
| Sdhc | down | down | + | + |  |
| Sdhd | down | down | + | + | + |
| Sucla2 | down | down | + | + | + |
| Suclg1 | down | down | + | + |  |
| Suclg2 | down | down |  | + | + |

**Supplementary Table S3: List of mitochondrial biogenesis-related genes that are differentially expressed in kidneys from *Glis3-*KO2 and/or *Glis3*-Pax8Cre PND28 mice and contain a GLIS3, HNF1B, and/or NRF1 binding peak (indicated by +) within +/-5kb from the corresponding TSS.**

| **Mitochondrial Biogenesis Genes** | | | | | |
| --- | --- | --- | --- | --- | --- |
| **gene symbol** | **PND28 *Glis3*-KO2** | **PND28 Pax8-Cre *Glis3*-KO2** | **GLIS3 Binding** | **HNF1B Binding** | **NRF1 Binding** |
| Acss2 | down | down | + | + | + |
| Alas1 | down | down | + | + | + |
| Apoo | down | down | + | + | + |
| Apool | down | down |  |  |  |
| Atf2 | down | unchanged | + | + | + |
| Atp5a1 | down | down |  |  | + |
| Atp5b | down | down |  |  | + |
| Atp5c1 | down | down | + | + | + |
| Atp5d | down | down | + | + | + |
| Atp5e | down | unchanged |  |  | + |
| Atp5f1 | down | down | + | + | + |
| Atp5g1 | down | down |  |  | + |
| Atp5g2 | down | down | + | + | + |
| Atp5g3 | down | down | + | + | + |
| Atp5h | down | down |  |  | + |
| Atp5j | down | down | + | + | + |
| Atp5j2 | down | down | + | + | + |
| Atp5k | down | down | + | + | + |
| Atp5l | down | down | + | + | + |
| Atp5o | down | down | + | + | + |
| Atp5s | down | down | + | + | + |
| Calm1 | up | unchanged | + | + | + |
| Camk4 | down | unchanged |  |  |  |
| Carm1 | unchanged | unchanged | + | + | + |
| Chchd3 | down | down |  |  | + |
| Chchd6 | unchanged | unchanged |  |  | + |
| Chd9 | unchanged | unchanged | + | + | + |
| Creb1 | down | unchanged | + | + | + |
| Crebbp | up | down | + | + | + |
| Crtc1 | unchanged | unchanged | + | + | + |
| Crtc2 | up | unchanged | + | + | + |
| Crtc3 | up | unchanged |  |  |  |
| Cycs | down | down | + | + | + |
| Dnajc11 | down | down |  |  | + |
| Esrra | down | down | + | + | + |
| Gabpa | unchanged | unchanged | + | + | + |
| Gabpb1 | unchanged | up | + | + | + |
| Glud1 | unchanged | down | + | + | + |
| Hcfc1 | unchanged | unchanged |  |  | + |
| Hdac3 | unchanged | unchanged |  |  | + |
| Helz2 | down | unchanged |  |  | + |
| Hspa9 | down | down | + | + | + |
| Idh2 | down | down | + | + |  |
| Immt | down | down | + | + | + |
| Mapk11 | up | up |  |  |  |
| Mapk12 | up | unchanged |  |  |  |
| Mapk14 | down | down | + | + | + |
| Med1 | unchanged | unchanged | + | + | + |
| Mef2c | unchanged | unchanged |  |  | + |
| Mef2d | up | unchanged | + | + | + |
| MICOS13 | down |  |  |  |  |
| Minos1 | down | unchanged | + | + | + |
| mt-Atp6 | unchanged | down |  |  |  |
| Mtx1 | down | down | + | + | + |
| Mtx2 | down | unchanged | + | + | + |
| Ncoa1 | unchanged | down |  |  |  |
| Ncoa2 | down | down | + | + | + |
| Ncoa6 | up | unchanged |  |  | + |
| Ncor1 | unchanged | down |  |  | + |
| Nr1d1 | down | down | + | + | + |
| Nrf1 | unchanged | unchanged | + | + | + |
| Perm1 | down | down |  |  |  |
| Polg2 | unchanged | down |  |  |  |
| Polrmt | down | down |  |  | + |
| Ppara | unchanged | down | + | + | + |
| Ppargc1a | down | down | + | + |  |
| Ppargc1b | down | down | + | + | + |
| Pprc1 | unchanged | up |  |  | + |
| Prkaa2 | down | down | + | + | + |
| Prkab1 | up | unchanged | + | + | + |
| Prkab2 | up | up | + | + | + |
| Prkag1 | unchanged | down | + | + |  |
| Prkag2 | up | unchanged | + | + | + |
| Prkag3 | down | down |  |  |  |
| Rxra | down | down | + | + |  |
| Samm50 | down | down |  |  | + |
| Sirt3 | down | down | + | + |  |
| Sirt4 | down | unchanged | + | + | + |
| Sirt5 | down | down | + | + | + |
| Smarcd3 | up | unchanged | + | + | + |
| Sod2 | down | down | + | + | + |
| Ssbp1 | down | unchanged | + | + | + |
| Tbl1x | up | unchanged | + | + | + |
| Tbl1xr1 | unchanged | down | + | + | + |
| Tfam | down | down | + | + | + |
| Tfb1m | unchanged | unchanged | + | + | + |
| Tfb2m | down | down | + | + | + |
| Tgs1 | up | up |  |  | + |
| Tmem11 | unchanged | down | + | + | + |
| Twnk | down | down |  |  | + |
| Usp46 | unchanged | unchanged | + | + | + |

**Supplementary Table S4:** **List of fatty acid oxidation-related genes that are differentially expressed in kidneys from *Glis3-*KO2 and/or *Glis3*-Pax8Cre PND28 mice and contain a GLIS3, HNF1B, and/or NRF1 binding peak (indicated by +) within +/-5kb from the corresponding TSS.**

| **Fatty Acid Oxidation** | | | | | |
| --- | --- | --- | --- | --- | --- |
| **gene symbol** | **PND28 *Glis3*-KO2** | **PND28 Pax8-Cre *Glis3*-KO2** | **GLIS3 Binding** | **HNF1B Binding** | **NRF1 Binding** |
| Abcd1 | unchanged | unchanged | + | + |  |
| Abcd2 | down | up |  |  |  |
| Abcd3 | down | down | + | + | + |
| Abcd4 | down | down | + | + | + |
| Acaa1a | down | down |  |  | + |
| Acaa2 | down | down | + | + | + |
| Acacb | down | down |  |  |  |
| Acad10 | down | down | + | + | + |
| Acad11 | down | down |  |  | + |
| Acadl | down | down | + | + | + |
| Acadm | down | down | + | + | + |
| Acads | down | down |  |  | + |
| Acadsb | down | down | + | + | + |
| Acadvl | down | down |  |  | + |
| Acat1 | down | down |  |  | + |
| Acat2 | down | down |  |  | + |
| Acot8 | down | down | + | + | + |
| Acox1 | down | down | + | + | + |
| Acox2 | down | unchanged |  |  |  |
| Acox3 | down | down | + | + | + |
| Acoxl | unchanged | down |  |  |  |
| Adipoq | unchanged | unchanged | + | + |  |
| Akt1 | up | up |  |  | + |
| Akt2 | unchanged | down | + | + | + |
| Amacr | down | down |  | + | + |
| Auh | down | down | + | + | + |
| Bdh2 | down | down | + | + | + |
| Cnr1 | up | up |  |  |  |
| Cpt1a | down | down | + | + | + |
| Cpt1c | up | up |  |  | + |
| Cpt2 | down | down | + | + | + |
| Crat | down | down | + | + | + |
| Crot | down | unchanged | + | + | + |
| Decr1 | down | down | + | + |  |
| Decr2 | down | down | + | + |  |
| Dld | down | down | + | + | + |
| Ech1 | down | down |  |  | + |
| Echdc1 | down | unchanged | + | + | + |
| Echdc2 | down | down |  |  |  |
| Echs1 | down | down | + | + |  |
| Eci1 | down | down | + | + | + |
| Eci2 | unchanged | down | + | + | + |
| Ehhadh | down | down |  |  |  |
| Etfa | down | down | + | + | + |
| Etfb | down | down |  |  | + |
| Etfdh | down | down |  |  | + |
| Fabp1 | down | unchanged |  |  |  |
| Gcdh | down | down |  |  |  |
| Hadh | down | down |  |  | + |
| Hadha | down | down | + | + | + |
| Hadhb | down | down | + | + | + |
| Hsd17b10 | down | down |  |  | + |
| Hsd17b4 | down | down |  |  | + |
| Irs1 | down | unchanged | + | + |  |
| Irs2 | up | unchanged | + | + | + |
| Ivd | down | down | + | + | + |
| Lep | up | up |  |  |  |
| Lonp2 | down | down | + | + | + |
| Mcat | down | down |  |  | + |
| Mettl20 | up | unchanged | + | + |  |
| Mfsd2a | down | unchanged |  |  | + |
| Mlycd | down | down | + | + | + |
| Pex2 | down | unchanged | + | + | + |
| Pex5 | down | down |  |  | + |
| Pex7 | down | down |  |  | + |
| Plin5 | down | down |  |  |  |
| Ppara | unchanged | down | + | + | + |
| Ppard | unchanged | unchanged | + | + | + |
| Scp2 | down | down | + | + | + |
| Sesn2 | up | down | + | + | + |
| Slc25a17 | down | down | + | + | + |
| Slc25a20 | down | down |  |  |  |
| Slc27a2 | down | down |  |  |  |
| Twist1 | up | unchanged |  |  |  |
| Tysnd1 | down | down |  | + | + |

**Supplementary Table S5. Primers for QPCR analysis.**

**Supplementary Methods**

**RNA-Seq and ChIP-Seq data analysis**

Raw RNA-seq reads were filtered remove fragments with mean base quality score less than 20. Filtered RNA-seq reads were mapped to the mm10 reference assembly via STAR v2.5 [1] with parameters "--outSAMattrIHstart 0 --outFilterType BySJout --alignSJoverhangMin 8 --limitBAMsortRAM 55000000000 --outSAMstrandField intronMotif --outFilterIntronMotifs RemoveNoncanonical". Counts per gene were determined via featureCounts (Subread v1.5.0-p1) [2]. Evaluated gene models are taken from GENCODE VM18 as downloaded from the UCSC Table Browser on December 17, 2018. Differential analysis was performed with DESeq2 v1.14.1 [3]. Gene expression heatmaps are based on rlog-transformed scores as reported by DESeq2 and generated by Graphpad Software. Association of mouse transcript identifiers with gene names was established using BioMart (as of July 5, 2018), followed by conversion to human orthologs using HCOP [4] (as of March 17, 2020) for use in GSEA. Pre-ranked gene lists for GSEA analysis were calculated using negative log10 adjusted p-values from DESeq2, multiple by -1 or +1 for down- or upregulation, respectively. Raw ChIP-seq reads were filtered remove fragments with mean base quality score less than 20. Adapters were removed by Cutadapt v1.12 [5] and filtered to retain only those with length at least 30nt. Reads were mapped against the mm10 reference assembly via Bowtie v1.2 [6], with only uniquely mapped hits accepted. Duplicate mapped reads were removed via MarkDuplicates.jar (using flag REMOVE_DUPLICATES=TRUE) from the Picard tool suite v1.110. Peak calls were made by HOMER (v4.10.3) [7] with parameters “-style factor”, comparing each ChIP sample against the respective input sample, then redefined as 300mers centered on the called peak midpoints.

**Gene set enrichment and pathway analysis**

Gene Set Enrichment Analysis (GSEA) software [8, 9] was used to analyze pre-ranked gene list from PND7, 14, and 28 *Glis3-KO2* vs WT RNA-Seq expression data. Pre-ranked gene lists were analyzed against GSEA’s hallmark and Gene Ontology (GO) gene set databases. The hallmark database was originally generated using a computational methodology to summarize and refine biological and cellular processes from the Molecular Signature Database (MSigDB) gene sets [10]. Hallmark datasets were ranked on GSEA analysis and scored by the normalized enrichment score (NES). A full list of GSEA negative enrichment hallmarks is included in Table S2. The GO ID, cellular component (CC), was used to identify the pre-ranked gene sets top cellular locations where the gene products performed a function [11, 12]. Functional enrichment analysis of downregulated RNA-Seq data (FDR <0.05) was based on Reactome Knowledgebase using Reactome web-based pathway analysis networks [13]. Differentially expressed genes were also analyzed with the mouse Mitocarta3.0 (https://www.broadinstitute.org/files/shared/metabolism/mitocarta/mouse.mitocarta3.0.html).

**Glis3 lentivirus**

The Glis3 lentiviral expression plasmid, pLVX-Glis3-mCherry, was derived from pLVX-mCherry-N1 (Clontech-Takarabio) as described previously [14, 15]. WT and *Glis3-KO2* primary kidney epithelial cells were transiently infected with pLVX-GLIS3-mCherry or pLVX-mCherry-N1 in 12-well plates and RNA was isolated 24 hr later for RT-qPCR analysis.

**RT‑qPCR analysis**

Total RNA was isolated from renal tissue or cultured renal primary cells using the PureLink RNA Mini Kit (ThermoFisher) according to the manufacturer’s protocol and cDNA was generated using a High-Capacity cDNA Reverse Transcription kit (Applied Biosystems). The generated cDNA was used with the Universal SYBR Green Supermix reagent (Applied Biosystems). The relative mRNA fold change in the expression of each gene was determined by the 2^− ΔΔCt^ method, and mouse *actin* primers were used as a reference for normalization. Primer sequences are listed in Supplementary Table S5.

**Mitochondria DNA copy number analysis**

The superior or inferior pole of the mouse kidney was cut and DNA isolated using the Qiagen DNeasy Blood and Tissue Kit (Qiagen) following manufacturers’ instructions. For qPCR 30 ng DNA was used to determine the relative mtDNA copy number. 16S and ND1 were selected for mitochondrial genes, as they are less prone to deletions, and the single copy β2-microglobulin was selected for the nuclear reference gene. Conditions of the thermocycler used were as described previously [16].

**Immunoblot analysis**

Protein was extracted from renal tissue or *in vitro* cell culture using RIPA assay buffer (ThermoFisher). Protease inhibitor cocktail (1:100) and phosphatase inhibitor cocktail (1:100) were added fresh before each extraction. Equal protein quantities (10–40 μg) were loaded onto 4–15% SDS-PAGE gels, resolved by gel electrophoresis, and transferred onto PVDF membranes (Bio-Rad). Membranes were blocked in 5% bovine serum albumin or 5% milk in TBST and incubated overnight with primary antibody at 4°C with gentle agitation. Primary antibodies used in these studies included TFB2M (ab118321; Abcam), OxPhos Cocktail (45-8099; ThermoFisher), and β-actin (MA5-15739; ThermoFisher). Membranes were incubated with the appropriate horseradish peroxidase-conjugated secondary antibody before visualization using SuperSignal West Femto enhanced chemiluminescence solution (34095; ThermoFisher) and the iBright FL 1000 (ThermoFisher). Optical density was determined using the ImageJ software from NIH and Image Studio Lite from LI-COR.

**Seahorse analysis**

Primary RECs were grown as described in Material and Methods and then trypsinized using TrypLE Express (12605010; ThermoFisher) and plated in 96-well Seahorse microplates for oxygen consumption rate (OCR) and glycolytic proton efflux rate (glycoPER) measurements at 1.8 x 10^4^ cells per well. We determined that this plating scheme did not affect the number of metabolically active primary RECs between WT and *Glis3*-KO2 using the PrestoBlue (A13261; Invitrogen) assay. The XF 96-well sensor cartridges were hydrated overnight in a CO_2_ free incubator at 37°C using sterile water. The sterile water was removed 60 min before loading of the injection ports on the sensor cartridge and replaced with Seahorse XF calibrant solution. The 96-well microplate media was replaced with Seahorse XF media (103575; Agilent) containing 5 mM HEPES for 60-90 min prior to starting assay. Oligomycin (1.5 μM), carbonyl cyanide 4-(trifluoromethoxy)phenylhydrazone (FCCP; 3 μM), and a combination of rotenone and antimycin A (0.5 μM each) or 2-deoxy-D-glucose (2-DG; 50mM) were loaded into the ports to be inject at specified times using a Seahorse XFe96 analyzer.

**Metabolomics sample processing**

Tissue samples were weighed and placed into 2 ml soft tissue homogenization tubes pre-filled with 1.4 mm ceramic beads (Omni International). HPLC-grade water (Fisher Chemical) was added to each to tube at the ratio of 50 mg of tissue to 1 ml of water. Homogenization was performed on bead mill homogenizer (Bead Ruptor Elite, Omni International) for 30 s on 2.6 m s^-1^. Visual inspection was performed to ensure complete homogenization. Three hundred µl of homogenate was placed into a 1.5 ml microcentrifuge tube and centrifuged (14,000 relative centrifugal force (rcf), 10 min, and 4°C). A processing blank was created by completing all sample processing steps including bead homogenization substituting tissue with HPLC-grade water. Two hundred µl of supernatant was transferred to a 1.5 ml microcentrifuge tube and 800 µl of ice-cold methanol ((Fisher Chemical, Optima^TM^ LC-MS grade) was added to each tube. The final methanol-water solution (4:1) *v/v* was placed in a -20ºC freezer for 30 min to enhance protein precipitation. Samples were centrifuged at 14,000 rcf for 10 min at 4°C. 750 µl of supernatant was transferred into a 1.5 ml microcentrifuge tube and dried via centrifugal evaporation (Genevac EZ-2 Plus, SP Scientific) on HPLC fraction mode with maximum heat at 40ºC. Samples were stored at -80 ºC until analysis. Dried extracts were removed from storage at -80°C, allowed to warm to room temperature, and resuspended via the addition of 250 microliters of acetonitrile-water (2%:98%) *v/v* to each sample, vortexed for 3 s, and centrifuged at 14,000 rcf for 10 min at 4ºC. 50 µl of each sample was combined into a pooled quality control (QC). An additional aliquot of each extract was transferred to a 2 ml autosampler vial with microvolume insert (Agilent). System blanks were generated by placing acetonitrile-water (2%:98%) *v/v* into autosampler vials.

**Metabolomics**

Untargeted metabolomics was performed on homogenates of kidneys from WT and *Glis3-KO2* mice at PND28 (extracts derived after cold-organic solvent protein precipitation) using an ultra-high performance liquid chromatograph (Vanquish, Thermo Scientific) coupled to a high-resolution mass spectrometer (Orbitrap Fusion Tribrid, Thermo Scientific). LC-MS and LC-MS/MS data were acquired. LC-MS data were collected from individual samples (n = 1 injection, volume = 5 µl), system blanks (injection of solvent used to resolubilize samples), and a pooled quality control. The pooled quality control (QC) was injected multiple times at different volumes and used in data processing. LC-MS/MS data, used to annotate features, were collected using the AcquireX (Thermo Scientific) deep scan methodology in which pooled QC was injected multiple times (n = 7). Compound Discoverer 3.3.0.550 (ThermoFisher) was used to process .raw files which resulted in a tabular output which included descriptors of each feature (e.g., *m/z*, retention time), annotation information (e.g., MS/MS database match), and peak area. We processed the output from Compound Discoverer using in-house R scripts via JupyterNotebooks. The major components of the processing included formatting of the data outputs, comparison of *m/z* and retention time of annotation features versus an in-house generated list based on authentic chemical standards, assessment of signal response in pooled QC samples, assessment of signal variance in pooled QC samples versus samples (i.e., dispersion ratio), and multi- and univariate statistics. Glutamine, glutamic acid, citric acid, and alpha-ketoglutaric acid were annotated in the data processing workflow or manually annotated. We affirmed the annotation of glutamine (241|145.0622|0.478), glutamic acid (242|146.04613|0.487), and citric acid (68|191.01967|0.685) in the negative ion mode to level 1 confidence based on MS/MS matching, accurate mass measurement, and retention time similarity to authentic chemical standard. Alpha-ketoglutaric acid was manually annotated (388|145.0145|0.652) using accurate mass measurement and manual interpretation of MS/MS product ion scan.

**TEM Sample Processing**

Three WT and three Glis3-KO2 kidney samples were placed in Modified Karnovsky fixative at necropsy. Processing of the samples was performed on 18-Mar-2020 in accordance with SOP 6.0.0, using a Leica EM TP processor. The samples were rinsed with buffer, post-fixed in 1% osmium tetroxide in phosphate buffer, rinsed in distilled water, dehydrated through an ethanol series, and transitioned to acetone. The samples were then infiltrated with increasing concentrations of Poly Bed 812 resin, embedded in pure Poly Bed 812, and placed in an oven to polymerize. Once polymerized, selected blocks were trimmed and semithin sections approximately 0.5 µm thick were cut, mounted on glass slides, and stained with 1% toluidine blue O in 1% sodium borate per SOP 9.0. The sections were examined by light microscopy to identify the areas of interest. Each block was initially trimmed to the area of interest, sectioned at approximately 90 nm thick, placed on 200 mesh copper grids, and stained with uranyl acetate and lead citrate per SOP 9.0. For this report, each block was recut at approximately 90 nm, placed on formvar-coated slotted grids, and stained as previously described. Digital images were captured with an AMT 16-megapixel mid-mount camera attached to a JEOL JEM-1400+ transmission electron microscope operating at an accelerating voltage of 80kV.

**Histology staining**

WT and *Glis3-KO2* kidneys at PND7, PND14, and PND28 were fixed in 10% neutral buffered formalin for 24 h, subsequently transferred to 70% ethanol, processed, embedded in paraffin, sectioned at 5 μm to visualize renal cysts.

**Lactate, glutamine, and citrate measurements**

WT and *Glis3-KO2* kidney lactate levels were measured using Lactate-Glo assay kit (J5021; Promega) according to manufacturer’s instructions. For WT and *Glis3-KO2* RECs lactate was measured in the media after a PBS washout and complete media replaced for 2 hours. For glutamine assay WT and *Glis3-KO2* RECs were washed with PBS and replaced with advanced DMEM/F-12 flex media containing only 3mM glutamine for 1 hour. Glutamine colorimetric assay kit (ab197011; Abcam), was used to measure glutamine concentration from the media according to manufacturer instructions. For the citrate assay WT and *Glis3-KO2* RECs were thoroughly washed with PBS and replaced with media containing either 10mM glucose or 3mM glutamine for 2 hours followed by measuring citrate concentration in cells using citrate assay kit (MAK333-1; Sigma-Aldrich) according to the manufacturer’s instructions.

**References**

[1] Dobin A, Davis CA, Schlesinger F, Drenkow J, Zaleski C, Jha S, et al. STAR: ultrafast universal RNA-seq aligner. Bioinformatics. 2013;29:15-21: <https://doi.org/10.1093/bioinformatics/bts635>.

[2] Liao Y, Smyth GK, Shi W. featureCounts: an efficient general purpose program for assigning sequence reads to genomic features. Bioinformatics. 2014;30:923-30: <https://doi.org/10.1093/bioinformatics/btt656>.

[3] Love MI, Huber W, Anders S. Moderated estimation of fold change and dispersion for RNA-seq data with DESeq2. Genome Biol. 2014;15:550: <https://doi.org/10.1186/s13059-014-0550-8>.

[4] Braschi B, Denny P, Gray K, Jones T, Seal R, Tweedie S, et al. Genenames.org: the HGNC and VGNC resources in 2019. Nucleic Acids Res. 2019;47:D786-D92: <https://doi.org/10.1093/nar/gky930>.

[5] Martin M. Cutadapt removes adapter sequences from high-throughput sequencing reads. EMBnetjournal. 2011;17:10-2: <https://doi.org/10.14806/ej.17.1.200>.

[6] Langmead B, Trapnell C, Pop M, Salzberg SL. Ultrafast and memory-efficient alignment of short DNA sequences to the human genome. Genome Biol. 2009;10:R25: <https://doi.org/10.1186/gb-2009-10-3-r25>.

[7] Heinz S, Benner C, Spann N, Bertolino E, Lin YC, Laslo P, et al. Simple combinations of lineage-determining transcription factors prime cis-regulatory elements required for macrophage and B cell identities. Mol Cell. 2010;38:576-89: <https://doi.org/10.1016/j.molcel.2010.05.004>.

[8] Subramanian A, Tamayo P, Mootha VK, Mukherjee S, Ebert BL, Gillette MA, et al. Gene set enrichment analysis: a knowledge-based approach for interpreting genome-wide expression profiles. Proc Natl Acad Sci U S A. 2005;102:15545-50: <https://doi.org/10.1073/pnas.0506580102>.

[9] Mootha VK, Lindgren CM, Eriksson KF, Subramanian A, Sihag S, Lehar J, et al. PGC-1alpha-responsive genes involved in oxidative phosphorylation are coordinately downregulated in human diabetes. Nat Genet. 2003;34:267-73: <https://doi.org/10.1038/ng1180>.

[10] Liberzon A, Birger C, Thorvaldsdottir H, Ghandi M, Mesirov JP, Tamayo P. The Molecular Signatures Database (MSigDB) hallmark gene set collection. Cell Syst. 2015;1:417-25: <https://doi.org/10.1016/j.cels.2015.12.004>.

[11] Ashburner M, Ball CA, Blake JA, Botstein D, Butler H, Cherry JM, et al. Gene ontology: tool for the unification of biology. The Gene Ontology Consortium. Nat Genet. 2000;25:25-9: <https://doi.org/10.1038/75556>.

[12] The Gene Ontology C. The Gene Ontology Resource: 20 years and still GOing strong. Nucleic Acids Res. 2019;47:D330-D8: <https://doi.org/10.1093/nar/gky1055>.

[13] Jassal B, Matthews L, Viteri G, Gong C, Lorente P, Fabregat A, et al. The reactome pathway knowledgebase. Nucleic Acids Res. 2020;48:D498-D503: <https://doi.org/10.1093/nar/gkz1031>.

[14] Kim YS, Kang HS, Takeda Y, Hom L, Song HY, Jensen J, et al. Glis3 regulates neurogenin 3 expression in pancreatic beta-cells and interacts with its activator, Hnf6. Mol Cells. 2012;34:193-200: <https://doi.org/10.1007/s10059-012-0109-z>.

[15] Takeda Y, Kang HS, Freudenberg J, DeGraff LM, Jothi R, Jetten AM. Retinoic acid-related orphan receptor gamma (RORgamma): a novel participant in the diurnal regulation of hepatic gluconeogenesis and insulin sensitivity. PLoS Genet. 2014;10:e1004331: <https://doi.org/10.1371/journal.pgen.1004331>.

[16] Quiros PM, Goyal A, Jha P, Auwerx J. Analysis of mtDNA/nDNA Ratio in Mice. Curr Protoc Mouse Biol. 2017;7:47-54: <https://doi.org/10.1002/cpmo.21>.
